# Transient protein expression in tobacco BY-2 plant cell packs using single and multi-cassette replicating vectors

**DOI:** 10.1007/s00299-020-02544-w

**Published:** 2020-04-24

**Authors:** Zuzana Poborilova, Helena Plchova, Noemi Cerovska, Cornelius J. Gunter, Inga I. Hitzeroth, Edward P. Rybicki, Tomas Moravec

**Affiliations:** 1 Institute of Experimental Botany of the Czech Academy of Sciences, Prague, Czech Republic; 2 Biopharming Research Unit, Department of Molecular and Cell Biology, University of Cape Town, Cape Town, South Africa

**Keywords:** Tobacco BY-2 cells, Transient co-expression, Plant cell pack, GFP, DsRed, Plant expression vector

## Abstract

**Key message:**

This is the first evidence that replicating vectors can be successfully used for transient protein expression in BY-2 plant cell packs.

**Abstract:**

Transient recombinant protein expression in plants and recently also plant cell cultures are of increasing interest due to the speed, safety and scalability of the process. Currently, studies are focussing on the design of plant virus-derived vectors to achieve higher amounts of transiently expressed proteins in these systems. Here we designed and tested replicating single and multi-cassette vectors that combine elements for enhanced replication and hypertranslation, and assessed their ability to express and particularly co-express proteins by *Agrobacterium*-mediated transient expression in tobacco BY-2 plant cell packs. Substantial yields of green and red fluorescent proteins of up to ~ 700 ng/g fresh mass were detected in the plant cells along with position-dependent expression. This is the first evidence of the ability of replicating vectors to transiently express proteins in BY-2 plant cell packs.

**Electronic supplementary material:**

The online version of this article (10.1007/s00299-020-02544-w) contains supplementary material, which is available to authorized users.

## Introduction

Generally, the expression of recombinant proteins in plants and plant cell cultures can be achieved either by developing stable transgenic lines or transient expression systems. One of the limitations to exploit exclusively stable transgenic systems for recombinant protein production is the length of time—ranging from months to years—required for their development (Sukenik et al. 2018; Yao et al. 2015). This problem has been overcome with the introduction of transient expression methods for whole-plant expression systems, where *Agrobacterium*-mediated delivery of vector DNA into most of the cells of a plant results in rapid and often high-level production of recombinant proteins (Rybicki 2010).

Plant cell culture is a well-established technology platform that has been used over 30 years to produce natural compounds for the biomedical, food and cosmetic industries (Wilson and Roberts 2012). Therefore, it is not surprising that this technology has been intensely studied in terms of its use for recombinant protein production. Although whole plants are more commonly used as plant-based production platforms via stable or transient expression, a number of studies involving plant cell suspension cultures as the production host have been published (Doran 2000; Hellwig et al. 2004; Tekoah et al. 2015). As with other eukaryotic expression systems, plant cells allow for post-transcriptional modifications of RNA and post-translational modifications of the protein of interest. Unlike bacterial and mammalian cells, plant cells are well suited for safe production of foreign proteins, as they are not a source of pathogens harmful to humans. One of the properties that favours the use of plant cell cultures over whole plants is the fact that cultivation takes place under fully controlled sterile conditions in chemically defined media (Häkkinen et al. 2018), which allows the production of recombinant proteins according to the current good manufacturing practice (Fischer et al. 2005). The high-value product can also be more easily recovered and purified, especially when the product is secreted into the culture medium (Fischer et al. 1999).

Lower yield of transiently expressed proteins from whole plants, or plant cells, is still a limiting factor for plant-based expression systems to be commercially adapted as an alternative production platform when compared to that of established expression systems (James and Lee 2001; Schillberg et al. 2019). Although recombinant antibody yields up to 2 g/kg have recently been achieved after transient expression in *Nicotiana benthamiana* leaves (Zischewski et al. 2016), this is still lower than the reported yields of 5–10 g/L routinely achieved in mammalian cell cultures (Schillberg et al. 2019).

Several approaches have been applied to enhance the production of recombinant proteins in cultured plant cells, including the modification of culture media components (Holland et al. 2010; James et al. 2000; Lee et al. 2002; Lee and Kim 2002; Wahl et al. 1995), cultivation conditions (Tsoi and Doran 2002) or the cultivation process (Raven et al. 2016). Secretion of the protein into the culture medium has so far been the preferred approach, as the secreted proteins can be recovered from the culture supernatant following free diffusion from the apoplast without any cell disruption (Fischer et al. 2005; Xu et al. 2011). The latest approach, which utilizes tobacco BY-2 cells in the form of three-dimensional (3D) medium-deprived porous plant cell aggregates (so-called plant cell packs—PCP), is a medium-free method: no medium supplements to either enhance protein stability or reduce protein degradation are required. In addition, the method itself has been shown to be very efficient and very quick, with the expression levels after a few days of various recombinant proteins in the range of 50–100 mg/kg (Rademacher et al. 2019).

Transient expression systems utilising virus-based vectors have the advantage of rapid and high-level expression of foreign proteins within a few days, compared to non-replicating systems (Yamamoto et al. 2018). Several plant virus-derived expression vector systems have been designed, and the most efficient ones described to date are based on DNA viruses, specifically geminiviruses (Hefferon 2014; Rybicki and Martin 2014), or single-stranded positive-sense RNA viruses, such as tobamoviruses, potexviruses and comoviruses (Hefferon 2017). *Bean yellow dwarf virus* (BeYDV), a geminivirus in the genus *Mastrevirus*, has a single-stranded circular DNA genome that can replicate to a very high copy-number as a plasmid-like dsDNA form via a rolling circle replication mechanism in the plant cell nucleus. BeYDV-derived expression vectors have been created by replacing the capsid protein (CP) and movement protein (MP) encoding sequences with a transgene expression cassette (Chen et al. 2011). One of the BeYDV-based expression vectors, pRIC, additionally includes the BeYDV replication associated elements (*rep*) *in cis* (Regnard et al. 2010). The vector DNA excises itself as a smaller replicon from the tumor-inducing (Ti) DNA of *Agrobacterium tumefaciens* by Rep-mediated rolling circle release. The pRIC expression system was used in *Nicotiana benthamiana* plants to generate *Human papillomavirus* type16 (HPV-16) L1 capsid protein as well as *Human immunodeficiency virus-1* type C p24 antigen derived from the Gag protein at yields up to ten-fold higher than a non-replicating form of the vector, and yields up to 1 mg/g fresh leaf tissue were obtained in another study that used a similar vector (Lai et al. 2012). The BeYDV-derived pRIC and similar vectors are also suitable for co-expression strategies, unlike the ssRNA virus-derived vectors, because of a lack of superinfection exclusion of other genomes (Rybicki and Martin 2014).

In this paper, we extend our previous work on transient expression in plants using plant virus-based vectors (Cerovska et al. 2008, 2013; Vaculik et al. 2015). Here, we focus on both transient expression and co-expression of two proteins in BY-2 cells, which had earlier been described in whole plants (Chen et al. 2011; Huang et al. 2010; Montague et al. 2011; Sainsbury et al. 2009). We chose to use the PCP technology (Rademacher et al. 2019), combining the advantages of fast-growing cell suspension cultures (specific growth rate up to 0.044/h for tobacco cell lines) (Xu et al. 2011) with those of transient protein expression assays to study the efficiency of replicating expression vectors derived from the pRIC vector (Regnard et al. 2010). In this work, we designed new vectors which also contain 5′/3′-untranslated regions (UTRs) from *Cowpea mosaic virus* (CPMV) which are present in the enhanced pEAQ expression vector (Sainsbury and Lomonossoff 2008): these contribute to the stability and hypertranslability of RNA transcripts (Chen et al. 2011; Huang et al. 2010; Montague et al. 2011; Sainsbury et al. 2009). Our new vectors containing identical expression cassettes were evaluated in terms of their ability to express and especially co-express the two reporter proteins, green fluorescent protein (GFP) and red fluorescent protein (DsRed), in tobacco BY-2 cells using the PCP technology.

## Material and methods

### Plant cell cultures

*Nicotiana tabacum* L. cv. Bright Yellow 2 (BY-2) suspension cells were grown in 250-mL Erlenmeyer flasks in liquid medium according to Murashige and Skoog (1962), modified by Nagata et al. (1992) in the dark at 26 °C on a rotary shaker (150 rpm). Cells were subcultivated once a week by transferring 2% (v/v) inoculum into fresh medium.

### Plant expression vectors

**Fig. 1:**
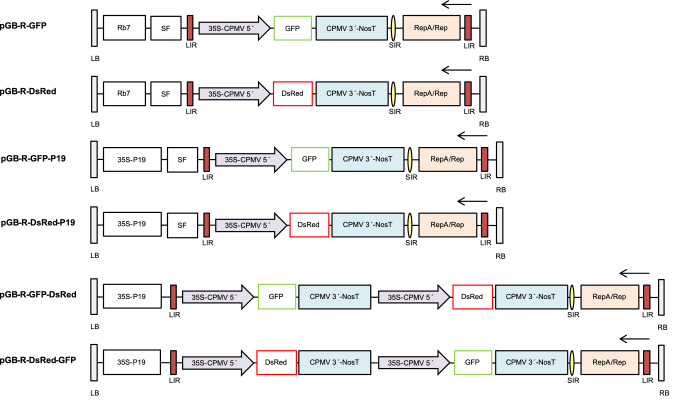
Schematic diagram of the T-DNA regions of the vectors used in this study. Rb7, *Nicotiana tabacum* Rb7 matrix attachment region; SF, short stuffer fragment of 35 bp; LIR, the long intergenic region from BeYDV; 35S-CPMV 5′, the 35S promoter from *Cauliflower mosaic virus* fused to 5′-UTR from *Cowpea mosaic virus*; GFP, green fluorescent protein; DsRed, *Discosoma* sp. red fluorescent protein; CPMV 3′-NosT, the nopaline synthase terminator from *Agrobacterium* fused to 3′-UTR from *Cowpea mosaic virus*; SIR, the short intergenic region from BeYDV; RepA/Rep, BeYDV ORFs encoding replication initiation protein (Rep) and RepA; 35S-P19, the expression cassette for the RNA silencing suppressor P19 consisting of the 35S promoter, the *Tobacco mosaic virus* omega leader sequence and the Nos terminator; LB and RB, the left and right borders of the T-DNA region

The expression vectors pGB-R (pRIC derivatives) were assembled using extended GoldenBraid 2.0 cloning strategy (Sarrion-Perdigones et al. 2013). Multigenic DNA constructs were created using DNA parts from GoldenBraid 2.0 kit, from Diego Orzaez (Addgene kit # 1000000076, basic plasmids, P19 ORF, Nos terminator), MoClo Toolkit, from Sylvestre Marillonnet (Addgene kit # 1000000044, 35S promoter and reporter genes), and using parts prepared at Laboratory of Virology IEB, Prague (MAR sequences, extended alpha 11–14 entry plasmids, domestication of pEAQ derived 5´/3´-UTR regions) and in the BRU, Cape Town (domesticated BeYDV sequences). The cloning was performed using type IIS restriction enzymes *BsmB*I, *Bsa*I and T4 DNA ligase (ThermoFisher Scientific, USA). In all assemblies, restriction–ligation reactions were carried out following previously described GB protocols (Sarrion-Perdigones et al. 2013). The GB entry vectors (pUPD1, pUPD2) and destination vectors (pDGB1α, pDGB3Ω) were used. All GB parts were verified by sequencing. The sequences of multi-cassette expression vectors are included in List S1. Schematic diagrams of the T-DNA regions of the plasmids pGB-R-GFP, pGB-R-GFP-P19, pGB-R-DsRed, pGB-R-DsRed-P19, pGB-R-GFP-DsRed and pGB-R-DsRed-GFP are shown in Fig. 1. The resulting plasmids were transformed into *Escherichia coli* (Top10, Thermo Fischer Scientific), and after isolation and sequence verification they were eventually transformed into *Agrobacterium tumefaciens* (EHA 105) by the freeze–thaw method (An 1987).

### *Agrobacterium* suspension

Recombinant *A. tumefaciens* (EHA 105) were used to transfect the BY-2 cells. *Agrobacterium* were grown for 2 days in a Luria–Bertani (LB) medium supplemented with antibiotics (0.1 mg/mL spectinomycin and 0.1 mg/mL rifampicin) to an optical density (OD)_600_ of approximately 1.0–1.2 with constant shaking at 180 rpm at 26 °C. Bacterial cells were harvested by centrifugation at 5000*g* for 6 min in a tabletop centrifuge at room temperature and resuspended in a sterile infiltration medium (Murashige and Skoog (MS) medium with 3% (w/v) sucrose supplemented with 0.2 mM acetosyringone to a final (OD)_600_ of 0.25. The bacterial suspensions were incubated for 2 h at 22 °C without shaking before applying onto the BY-2 cells.

### Transient expression in tobacco BY-2 cells

The recently developed expression system, which is based on the creation of 3D culture medium deprived porous plant cell aggregate, was chosen to transiently express fluorescent proteins in the BY-2 cultured cells (Rademacher et al. 2019). Briefly, 4-day-old BY-2 cultured cells (100 mL) with a packed cell volume (PCV) of 50% (v/v) were dispensed under sterile conditions into syringes equipped with a cellulose filter followed by the removal of the excess medium by application of 70 kPa vacuum using a Vac-Man vacuum manifold (Promega, USA) and vacuum pump Rocker 811 (Rocker Scientific, Taiwan), resulting in the formation of porous medium-free plant cell packs (PCP) or “cookies”. These PCPs were completely submerged with an excess volume of *Agrobacterium* suspension and incubated for 30 min at 22 °C. The excess liquid was then completely removed by vacuum filtration. The infused PCPs were then incubated for 4 days at 26 °C in the dark. All samples were analyzed in triplicate and three independent experiments were performed.

### Fluorescence imaging

The GFP and DsRed fluorescence imaging was performed by illuminating the PCPs under UV LED light (400 nm) and green LED light (530 nm), respectively. The fluorescence was observed and photographed through yellow (Roscolux 12: Straw for GFP) and red (Roscolux 19: Fire for DsRed) excitation filters with an Olympus digital camera (OLYMPUS PEN Lite E-PL3) and Zeiss Pancolar 50/1.8 lens. The pictures were taken 4 days after PCP infusion. The same procedure was used for GFP and DsRed fluorescence detection in *Agrobacterium* suspensions.

### Total RNA extraction, cDNA synthesis and quantitative RT-PCR (RT-qPCR)

BY-2 cells were mechanically disrupted using liquid nitrogen and a prechilled mortar and pestle. Total RNA was isolated using the Spectrum™ Plant Total RNA kit (Sigma Aldrich). Residual genomic DNA was removed using the DNA-free™ DNA removal kit (Invitrogen™). First-strand cDNA was synthesized from 1.0 µg of total RNA using M-MLV Reverse Transcriptase (Promega, USA) and oligo dT_23_ (Eurofins Genomics, Germany). All steps were performed following the manufacturer’s instructions. The cDNA was amplified in 96-well plate (Axygene, USA) using a LightCycler® 480 instrument, LightCycler® 480 SYBR Green I Master mix (Roche Life Science, Switzerland) and the following program: an initial denaturation at 95 °C for 10 min followed by 45 cycles of 10 s at 95 °C, 20 s at annealing temperature and 20 s at 72 °C for amplicon extension, followed by melting curve analysis. The reaction mixture consisted of master mix, 0.2 µM forward primer, 0.2 µM reverse primer, and cDNA at an equivalent to 20 ng RNA to the final volume of 10 µL. The following primers were used for *GFP* (FP: 5′-ACGTAAACGGCCACAAGTTC-3′, RP: 5′-AAGTCGTGCTGCTTCATGTG-3′), *DsRed* (FP: 5′-TATATGTCAAGCACCCTGCC-3′, RP: 5′-CCATCGGAAGGAAAGTTCAC-3′) and *EF-1α* (FP: 5′-TGAGATGCACCACGAAGCTC-3´, RP: 5′-CCAACATTGTCACCAGGAAGTG-3′). The GFP and DsRed mRNA levels were calculated as multiples of the *EF-1α* housekeeping gene from *N. tabacum* cv. BY-2 (Liu et al. 2012) using the 2^−ΔΔCt^ method. Threshold cycles and melting curves were calculated using the LightCycler® 480 Software (Version 1.5, Roche Life Science). RT-qPCR was conducted in triplicate for each biological replicate.

### Protein extraction and quantification

Total soluble protein (TSP) was extracted from 100 to 200 mg fresh mass of the infused PCPs and *Agrobacterium* cells harbouring the respective expression vectors. The cells were frozen in liquid nitrogen in microfuge tubes, ground by bead beating using FastPrep-24 Instrument (MP Biomedicals), dissolved in a double volume of extraction buffer (1 × phosphate-buffered saline; 137 mM NaCl, 2.7 mM KCl, 10 mM Na_2_HPO_4_, 1.8 mM KH_2_PO_4_, pH 7.4), 1 mM EDTA (Sigma Aldrich, St. Louis, MO, United States), 1 × protease inhibitor cocktail (ThermoFisher Scientific) (Häkkinen et al. 2018), and again subjected to homogenization using the FastPrep-24 Instrument. The extracts were centrifuged (10,000*g* at 4 °C) for 15 min and used for further analyses.

Extracted TSP was quantified using the Pierce™ BCA Protein Assay Kit (ThermoFisher Scientific). Twenty-five µL of 1:5 diluted extract and 200 µL of the working reagent were added to a transparent 96-well microplate (ThermoFisher Scientific) and incubated at 37 °C for 30 min. The absorbance was subsequently measured at 562 nm using a plate reader Infinite F200 PRO (Tecan, Austria). BSA standards in the range of 0.1–1.0 mg/mL were used to create a standard curve.

### Immunoblot analysis

Extracted TSP (10 µg) from both PCPs and *Agrobacterium* cells was loaded on 10% (w/v) sodium dodecyl sulphate–polyacrylamide gel (TGX™ FastCast™ Acrylamide Kit, Bio-Rad, USA), separated by electrophoresis, and transferred onto a nitrocellulose membrane (Whatman® Protran®, Sigma Aldrich). To specifically detect the presence of GFP and DsRed, the western blot was probed with the rabbit polyclonal anti-GFP antibody (ExBio, Czech Republic) at 1:1,000 dilution, and the rabbit polyclonal anti-RFP antibody (MBL International, USA) at 1:1,000 dilution. The secondary antibody used for the detection of both proteins was alkaline phosphatase-conjugated goat anti-rabbit IgG (H + L) (Sigma Aldrich) at 1:30,000 dilution. The blots were developed with 5-bromo-4-chloro-3-indolyl phosphate/nitro blue tetrazolium substrate tablets (Sigma Aldrich). The band intensities were quantified with the ImageJ software (Version 1.48). GFP (Abcam, United Kingdom) and DsRed (Fraunhofer IME, Germany) proteins in the range of 0.005–1.0 mg/mL were used to create the standard curves.

### GFP and DsRed fluorescence measurement

The degree of DsRed and GFP expression was also assessed via fluorescence spectroscopy. Fluorescence in the BY-2 extracts was measured using a plate reader (Infinite F200 PRO, Tecan, Austria) fitted with a 535/25 nm (excitation) and 589/25 nm (emission) filter set to quantify DsRed and a 485/25 nm (excitation) and 535/25 nm (emission) filter set for GFP quantification.

### PCR-based detection of circularized replicons

To detect the presence of circularized replicons in tobacco BY-2 cells, the extracts from infused PCPs were prepared. Frozen cells (100–200 mg fresh mass) were homogenized in a double volume of extraction buffer (1 × phosphate-buffered saline; 137 mM NaCl, 2.7 mM KCl, 10 mM Na_2_HPO_4_, 1.8 mM KH_2_PO_4_, pH 7.4), 1 mM EDTA (Sigma Aldrich, St. Louis, MO, United States) using a FastPrep-24 Instrument. The extracts were centrifuged (10,000*g* at 4 °C) for 15 min and used as a template for PCR. The oligonucleotides (FP: 5′-GAGCCACCTTCCTTTTCCAC-3′, RP: 5′-GAGCACTTGGGATAGGTAAG-3′) used in the PCR analysis were designed to amplify a 449 bp DNA fragment encompassing the sequence located upstream and downstream of LIR sequence in circularized replicons. The amplicons presence was verified by 1% (w/v) agarose gel electrophoresis run for 30 min at constant voltage 100 V (Bio-Rad, USA). GelRed-stained gel was photographed using a Molecular Imager®ChemiDoc^TM^XRS (BioRad, USA).

### Statistical analysis

Statistical analysis was performed using IBM SPSS Statistics (IBM, USA). The results are expressed as mean values ± SD. An independent *t *test was performed to determine the significant difference between the groups. Values *P* < 0.05 were considered statistically significant.

## Results

### Transient GFP and DsRed expression/co-expression in BY-2 cell packs

**Fig. 2:**
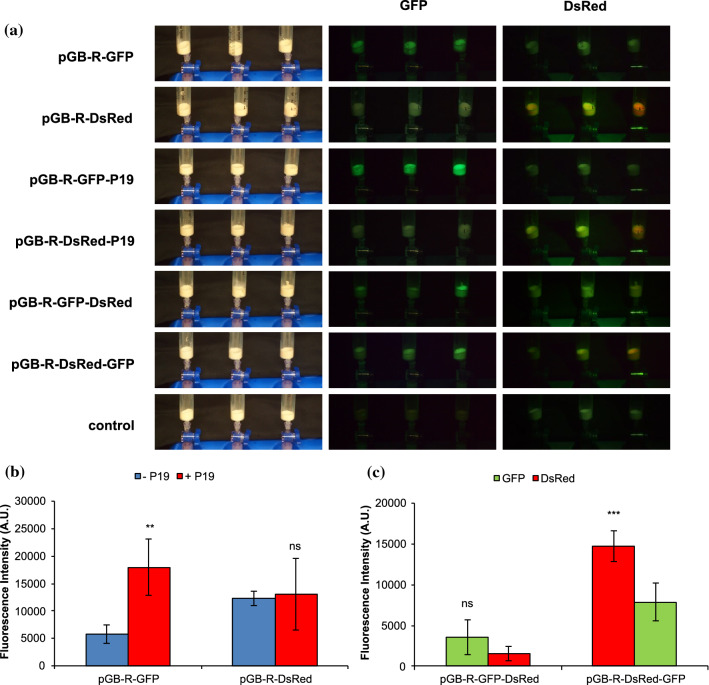
Transient GFP and DsRed expression/co-expression in the BY-2 cell packs induced by pGB-R expression vectors. **a** Expression of GFP (middle column) and DsRed (right column) protein detected in the BY-2 cells illuminated by UV and green light and observed under the yellow and red emission filter, respectively. Data displayed on the left side represent the vectors used. Representative photos of control and fluorescent proteins expressing BY-2 cells were chosen. **b, c** The values of GFP and DsRed fluorescence (A.U.) in the BY-2 cells shown as average ± standard deviation (SD) of three independent replicates. Significant difference was evaluated between pGB-R-GFP/pGB-R-DsRed vector without and with P19 (**b**) and between the individual expression cassettes within the multiple cassette vector (**c**) where the position-dependent expression was observed. The data were analyzed by *t*-test (*P* < 0.05). ns means no significant difference, **correspond to a significant difference at *P* < 0.01, *** correspond to a significant difference at *P* < 0.001

In this study, the ability of pGB-R replicating vectors to transiently express foreign proteins in tobacco BY-2 cell packs was investigated (Fig. 1). First, the expression of fluorescent proteins from replicating pGB-R vectors containing either GFP (pGB-R-GFP) or DsRed (pGB-R-DsRed) expression cassette, alternatively combined with P19 (pGB-R-GFP-P19 and pGB-R-DsRed-P19), was compared. Both GFP and DsRed fluorescence was observed in the PCPs illuminated by UV or green light, respectively (Fig. 2a, middle and right column). No DsRed fluorescence was observable in the cell packs infused by *Agrobacterium* harbouring pGB-R-GFP and pGB-R-GFP-P19 expression vectors and vice versa, no GFP fluorescence was noticed when pGB-R-DsRed or pGB-R-DsRed-P19 vectors were applied. Likewise, no green or red fluorescence was detected in control cell packs infused with MS medium only. Similarly, the *Agrobacterium* suspension itself did not show any GFP and DsRed fluorescence (Fig. S1). It was noticed that the fluorescence intensity peaked at 4 days after the PCP infusion and declined afterwards (not shown), therefore it was quantified at 4 days after infusion. The pGB-R-GFP vector resulted in approximately threefold lower fluorescence when compared to the pGB-R-GFP-P19. On the other hand, the P19 showed no effect on DsRed fluorescence when expressed from pGB-R-DsRed-P19 compared to pGB-R-DsRed (Fig. 2b).

Second, the co-expression of GFP and DsRed from pGB-R-GFP-DsRed and pGB-R-DsRed-GFP multi-cassette vectors was studied. It was found that the vectors induced transient expression of both fluorescent proteins (Fig. 2a, middle and right column), although this was slightly lower for each compared to the above mentioned pGB-R vectors expressing only GFP or DsRed protein. Interestingly, the fluorescence intensity measurement revealed the co-expression to be position-dependent within the individual multi-cassette vectors (Fig. 2c): this was subsequently studied in detail in relation to protein yields. While use of pGB-R-GFP-DsRed resulted in no significant difference between GFP and DsRed fluorescence intensity, a relationship between the fluorescence intensity and the expression cassette position within the vector was observed for pGB-R-DsRed-GFP vector. Here, the first expression cassette drove significantly higher protein expression (1.8 times) than the second one.

### Replicons formation in the BY-2 cells

**Fig. 3:**
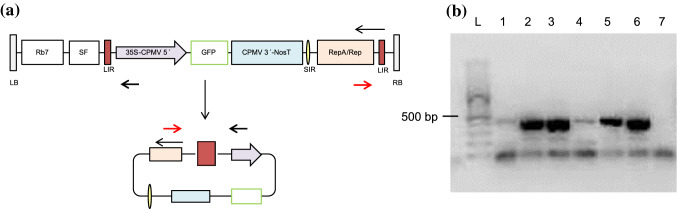
Presence of circularized replicons in tobacco BY-2 cells. **a** Scheme of T-DNA region of pGB-R-GFP expression vector illustrating a linear and a circularized form. Black and red arrow indicates primer positions. **b** Detection of a 449 bp PCR product confirming the presence of replicons in the BY-2 cells. Lanes are marked L, Gene Ruler 100 bp DNA Ladder (Life Technologies); 1, pGB-R-GFP; 2, pGB-R-DsRed; 3, pGB-R-GFP-P19; 4, pGB-R-DsRed-P19; 5, pGB-R-GFP-DsRed; 6, pGB-R-DsRed-GFP; negative control (wild type BY-2)

The formation of replicons in tobacco BY-2 cells was confirmed by PCR. The amplicons are created only when BeYDV Rep acts on *Agrobacterium*-delivered linear T-DNA inside cell nuclei to initiate rolling circle generation of linear ssDNA bounded by two flanking LIR sequences, that is then circularized and ligated by Rep, and converted to dsDNA by host polymerases (Fig. 3a). PCR amplicons of 449 bp confirming replicon formation were detected in all BY-2 cells packs transformed with respective pGB-R expression vectors, but not in the medium-treated BY-2 cells used as a negative control (Fig. 3b).

### Detection and quantification of protein expression in plant cell packs

**Fig. 4:**
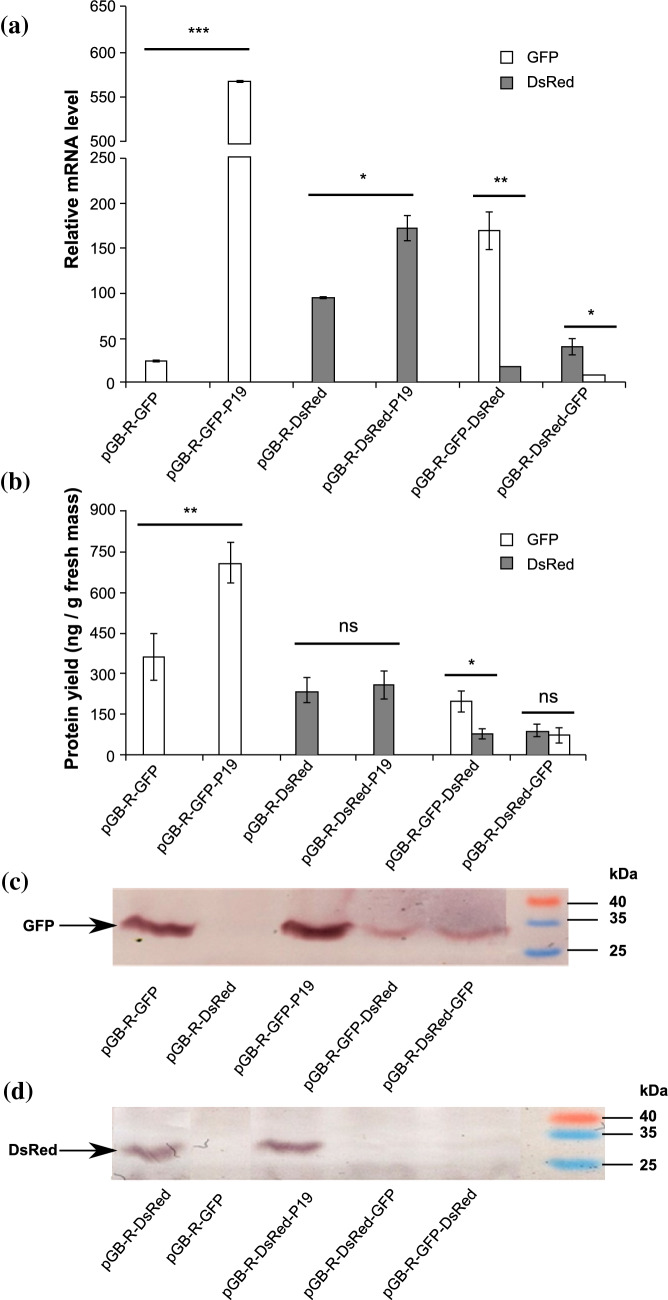
Detection of GFP and DsRed mRNA levels and protein expressions using RT-qPCR and Western blot analysis, respectively. **a** The mRNA expression level of GFP and DsRed from infused BY-2 cell packs presented as multiples of the *EF-1α* housekeeping gene mRNA. The values represent average ± standard deviation (SD) of three independent replicates. **b** The amounts of transiently expressed GFP and DsRed proteins from infused BY-2 cell packs. The values (ng/g of fresh mass) represent average ± standard deviation (SD) of three independent replicates. **c, d** Western blot analysis of GFP and DsRed proteins. The arrows indicate the bands corresponding to 27-kDa of GFP and 28-kDa of DsRed, respectively. The data were analyzed by *t *test (*P* < 0.05). ns means no significant difference, *correspond to a significant difference at *P* < 0.05, **correspond to a significant difference at *P* < 0.01, ***correspond to a significant difference at *P* < 0.001

The GFP and DsRed mRNA levels were measured using RT-qPCR to evaluate the effect of P19 on transgene expression and the position-dependent transgene co-expression. A similar pattern was observed for both GFP and DsRed mRNA expression, with differences between the respective expression vectors (Fig. 4a); the relative mRNA level from pGB-R-GFP-P19 and pGB-R-DsRed-P19 was approximately 23-fold and 1.8-fold higher than the one observed from pGB-R-GFP and pGB-R-DsRed, respectively. Comparison of GFP and DsRed mRNA levels within the individual multi-cassette vectors showed 8.5-fold higher mRNA level of GFP than DsRed in the case of pGB-R-GFP-DsRed vector and four-fold higher mRNA level of DsRed than GFP for pGB-R-DsRed-GFP vector. Furthermore, these increases were determined to be significant.

The yields of recombinant protein were in the range of 165–711 ng/g fresh mass, depending on the vector used (Fig. 4b). GFP yield was about two-fold higher from pGB-R-GFP-P19 in comparison to pGB-R-GFP, while DsRed protein accumulation was similar for both single cassette vectors pGB-R-DsRed and pGB-R-DsRed-P19. Multi-cassette vector pGB-R-GFP-DsRed resulted in 2.5-fold higher yield of GFP than that of DsRed; however, co-expression from pGB-R-DsRed-GFP did not show a significant difference between the abundance of DsRed and GFP. These findings are partially in line with mRNA expression levels (Fig. 4a), as lower GFP/DsRed mRNA levels were not necessarily coupled with lower protein yields and vice versa. For example, pGB-R-DsRed produced a 1.8-fold lower mRNA level than its counterpart containing P19, but the protein yields were similar for both vectors. Likewise, GFP mRNA level for the multi-cassette vector pGB-R-DsRed-GFP was four-fold lower than the one of DsRed, yet the protein yields were similar. In the case of pGB-R-GFP and pGB-R-GFP-P19 and the multi-cassette pGB-R-GFP-DsRed, however, the increase in mRNA levels coincided with the increase in protein levels.

The expression of fluorescent markers GFP and DsRed in the BY-2 cells was also analyzed by western blotting, with respective protein bands detected using specific antibodies against GFP and DsRed. The distinct single band of 27 kDa corresponding to GFP was detected when pGB-R-GFP construct was applied either with or without P19 co-expression (Fig. 4c). GFP bands of lower intensity were observed when pGB-R-GFP-DsRed and pGB-R-DsRed-GFP were used. Western blot analysis with anti-DsRed specific polyclonal antibody showed one distinct band of approximately 28 kDa corresponding to a monomeric DsRed (Fig. 4d). The single cassette expression vectors pGB-R-DsRed and pGB-R-DsRed-P19 showed distinctly higher expression of DsRed than the vectors pGB-R-DsRed-GFP and pGB-R-GFP-DsRed that co-express both target proteins, and for which bands of lower intensities were observed. To exclude possible GFP and DsRed expression from *Agrobacterium* cells harbouring the plasmids used in this study, the extracts from bacterial cells were also subjected to western blot analysis: no bands corresponding to GFP and DsRed were detected (Fig. S2).

## Discussion

Using plants and plant cell cultures for the production of pharmaceuticals has received much attention, and several plant-derived therapeutic proteins are either already available on the market, or in pre-clinical and clinical trials (Donini and Marusic 2019). A significant breakthrough was made in 2012, when the US Food and Drug Administration (FDA) approved for the first time a plant cell-derived enzyme, ELELYSO™ (taliglucerase alfa), for human use as a therapeutic for Gaucher disease (Yao et al. 2015). Transient plant expression systems have also been shown as a promising approach for addressing viral epidemics, by producing recombinant proteins on demand within only a few weeks for production-scale manufacture in the case of a pandemic influenza vaccine candidate by Medicago Inc. (Peyret and Lomonossoff 2015; Rybicki 2017; Yao et al. 2015). Plant virus expression vectors are becoming a powerful tool in plant-made biopharmaceutical development (Hefferon 2017). This, coupled with the recently developed technology of plant cell packs (Rademacher et al. 2019), allows scalable transient expression in cultured plant cells, enabling fast optimization of expression constructs and the possibility of obtaining amounts of recombinant proteins similar or higher to those expressed in stably transformed plants (Gengenbach et al. 2018).

In this study, replicating pGB-R vectors derived from the BeYDV-based expression vector pRIC (Regnard et al. 2010) were investigated in terms of their ability to transiently express and especially co-express foreign proteins in tobacco BY-2 cells, using the novel plant-cell pack technology. First, we tested the single cassette containing replicating vectors, pGB-R-GFP and pGB-R-DsRed, and their responsiveness to addition of RNA silencing suppressor P19. High levels of GFP and DsRed fluorescent protein expression both in presence and absence of P19 were observed within 4 days after PCP infusion (Figs. 2a, 4b). Similarly, in whole plants, the optimal yield of proteins expressed from the BeYDV vector is typically at 4 dpi (Hefferon 2014). Previously, the optimization of BeYDV-derived replicon vector system resulted in a rapid production of VLPs in *N. benthamiana* and the highest yield of transgenes (GFP and VLPs) was obtained within 5 days. Interestingly, co-delivery of a P19 expression cassette to this single replicon vector had no significant effect on the enhancement of the transgene expression (Huang et al. 2009). Our vectors pGB-R-GFP-P19 and pGB-R-DsRed-P19, both carrying the P19 expression cassette, resulted in strong fluorescence intensity with a significant increase in the case of pGB-R-GFP-P19 while there was no difference for pGB-R-DsRed-P19 (Fig. 2b). The importance of P19 for the enhanced protein expression by the BeYDV-derived vector was previously reported on *N. benthamiana* plants (Chen et al. 2011; Diamos and Mason 2019; Shah et al. 2013; Yamamoto et al. 2018). In addition, the impact of P19 on the protein production has been shown to be both synergistic and antagonistic, depending on particular plant species (Amiri et al. 2018; Angel et al. 2010; Garabagi et al. 2012). A marked discolouring of infiltrated *N. tabacum* leaves at 2–3 dpi leading to necrosis by 7 days (Angel et al. 2010), concomitant with a decrease in antibody production has been observed, likely due to cell death triggered by the hypersensitive response (HR) (Garabagi et al. 2012). Recently, the adverse effect of the P19 on the production of mutated tissue plasminogen activator 4 dpi has been described in the case of *N. benthamiana* plants, but not on *N. tabacum* cv. Xanthi plants (Amiri et al. 2018). As we did not observe any adverse impact of P19 on both the BY-2 PCPs and the transient expression of GFP and DsRed from pGB-R-GFP and pGB-R-DsRed vectors, the multi-cassette vectors designed for the co-expression of both proteins in tobacco BY-2 cells included the expression cassette for P19.

The BeYDV-based vectors have been shown suitable for co-expression strategies (Chen et al. 2011; Huang et al. 2010; Montague et al. 2011; Sainsbury et al. 2009). Generally, high yield efficient co-expression and assembly of multiple proteins can be achieved either by *Agrobacterium*-mediated co-delivery of more expression vectors, or by creating a single-vector system that contains multiple replicon cassettes. The latter strategy provides considerable simplification, does not create “competing replicons” with subsequent preferential amplification of one of the vectors in one cell, and is therefore to be preferred (Peyret and Lomonossoff 2015). Here we aimed to achieve co-expression from one-replicon systems, and created replicating pGB-R-GFP-DsRed and pGB-R-DsRed-GFP vectors that co-express GFP and DsRed from identical expression cassettes (Fig. 1). Both types of vectors induced transient expression of both fluorescent proteins in tobacco BY-2 cells as observed under UV and green illumination (Fig. 2a) and determined by fluorescence intensity measurement (Fig. 2c). Interestingly, the GFP and DsRed co-expression was found to be position-dependent with significantly higher protein expression detected from the first expression cassette (Fig. 4b). Efficient co-expression of two different proteins (GFP and DsRed) using a BeYDV-based vector containing two tandemly linked replicons was previously shown (Huang et al. 2010). Huang et al. confirmed that two replicons can efficiently replicate without any interference and both GFP and DsRed fluorescence were simultaneously detected in ~ 95% of *N. benthamiana* leaf cells. Each of the replicons was defined by LIR borders, and the placement of a single LIR between two replicons was shown to facilitate release and amplification of both replicons in the plant. The same dual replicon geminiviral vectors were successfully used to fully assemble therapeutic humanized mAbs 6D8 against Ebola and hE16 against West Nile virus in lettuce (*Lactuca sativa*) (Lai et al. 2012). No positional effect of expression cassettes on protein co-expression was described there, probably due to the fact that the two-replicon vector was used.

To elucidate a possible reason behind observed position-dependent protein co-expression, the levels of GFP and DsRed mRNAs were determined (Fig. 4a). RT-qPCR confirmed higher mRNA levels for the proteins expressed from the first expression cassette than from the second. We have also noticed that these higher mRNA levels did not entirely correspond to protein expression (Fig. 4b). Indeed, the high levels of mRNA do not have to necessarily correlate to high protein yields, as the number of mRNA molecules is not a limiting factor for translation (Jansing and Buyel 2019). Previously, the advantages of DNA replicons in terms of the insert size have been described (Palmer and Rybicki 1998) with no known size limit for geminiviral replicons compare to the RNA replicons, which become unstable with the larger inserts (Chen et al. 2011; Huang et al. 2009). Recently, however, Diamos and Mason (2019) described how larger BeYDV replicons accumulate to lower amounts than the smaller replicons and that the additional incorporation of 1.2 kb Rb7 MAR into the BeYDV vector may also reduce replicon accumulation. In our case, the total size of inserts ranges from 5.3 to 7.3 kb for single cassette and multiple cassette vectors, respectively. Thus, based on the above findings this complexity presumably affects the replicons’ behaviour and concomitant protein co-expression.

Combining key elements of the different expression systems, which are not mutually exclusive, could contribute to development of more effective systems. Recently, the advantages of pEAQ vectors, PVX-based vectors, and P24 silencing suppressor were successfully combined and resulted in a new vector, pEff, for transient expression of recombinant proteins in plants (Mardanova et al. 2017). Similarly, the combination of a P19 cassette with the CPMV- *hypertranslatable* (CPMV-*HT*) UTRs and a geminivirus-based LIR-SIR-LIR system represents another possible improvement (Peyret and Lomonossoff 2015). In fact, our pGB-R vectors are based on this suggestion, and the CaMV 35S promoter is fused with CPMV-5´UTR, Nos terminator with CPMV-3´UTR and the expression cassette is inserted between LIR and SIR of the BeYDV accompanied by a P19 cassette provided *in cis*. The pGB-R vectors thus combine the unique properties of a replicating geminiviral expression system with a non-replicating pEAQ-derived vector as they contain also CPMV-*HT* UTRs, unlike previously reported pRIC-type vectors (Diamos et al. 2016; Diamos and Mason 2019); therefore, they have the potential to drive both the efficient replication and hypertranslation of one or multiple GOIs from the same T-DNA (Peyret and Lomonossoff 2015).

We found pGB-R vectors to be efficient in the production of GPF and DsRed proteins in tobacco BY-2 cell packs. The single cassette vectors (pGB-R-GFP/DsRed and pGB-R-GFP/DsRed-P19) resulted in protein yields in the range of 260–711 ng/g fresh mass, while the total protein amount for the multi-cassette vector pGB-R-GFP-DsRed and pGB-R-DsRed-GFP was 277 ng/g fresh mass and 165 ng/g fresh mass, respectively (Fig. 4b). Previous studies employing the pRIC-based vectors for transient expression of various proteins in *N. benthamiana* plants have reported yields in the orders of μg to mg/g fresh leaf tissue, with the lowest yields of ~ 3–4 μg/g obtained for HIV-1 p24 (Regnard et al. 2010) and foot and mouth disease (FMD) VLPs (Ruiz et al. 2018), and the highest yields of 1.0–3.7 mg/g for the mAb rituximab, *Norwalk virus* capsid protein (NVCP) (Diamos et al. 2016), and GFP (Yamamoto et al. 2018). Using the pGB-R vectors, the obtained yields were lower than the minimum reported; however, these studies were performed on whole-plant models while we used cultured plant cells.

Only a few previous studies report transgenic or transient expression in plant cell suspensions using geminiviral replicons, with protein yields largely non-quantified. One early study used biolistically transformed tobacco NT1 cells with Rep expressed *in trans* off another vector to amplify β-glucuronidase (GUS) expression by 40-fold compared to a non-replicon vector (Mor et al. 2003). Shortly thereafter, NT1 cells biolistically transformed with a BeYDV-derived vector coding a staphylococcus enterotoxin B (SEB) gene, together with a plasmid with 35S-driven Rep, expressed SEB at a 20-fold higher level than when using a non-replicating vector (Hefferon and Fan 2004). Zhang and Mason (2006) used stable transgenic lines of tobacco NT1 cells with an ethanol-inducible BeYDV Rep-mediated replicon generator to express *Norwalk virus* capsid protein to a maximum of 12 ng/μg TSP. We note that our use of the latest medium-free plant cell pack generation and transformation methods are almost certainly easier and cheaper than use of biolistics or stable transformation for protein expression; moreover, expression levels appear higher, although comparisons of yields in ng or μg per μg TSP or packed cell fresh mass are rather unreliable.

To date, there are three publications describing the great potential of this method and comparing protein levels reached in PCPs and in whole plants (Gengenbach et al. 2018, 2019; Rademacher et al. 2019). The protein expression levels achieved using the PCP technology in these studies are comparable to those achieved in *N. benthamiana* leaves, depending on the proteins expressed, gene design or incubation time. We obtained lower protein amounts compared to these studied, which could be caused by the differences between the vectors used; our pGB-R vectors are the BeYDV-based replicating expression vectors and contain also CPMV-*HT* UTRs, while the *Agrobacterium* pTRA vector represents their non-replicating counterpart, and relies preferentially on the enhanced translation by the presence of tobacco Rb-7 scaffold attachment regions (Maclean et al. 2007) rather than amplification.

Our study describes the first evidence that replicating vectors can be successfully used for transient protein expression in BY-2 plant cell packs, and provides a useful toolkit for further exploration of this valuable new technology.

### Author contribution statement

ZP, HP, and TM conceived and directed this study; ZP, HP and TM designed the experiments; ZP analyzed the data and wrote the original draft manuscript; HP, NC, CJG, IIH, EPR and TM provided suggestions and revised the manuscript.

## Electronic supplementary material

Below is the link to the electronic supplementary material.

Supplementary file1 (DOCX 447 kb)
