# Supplementary material for: Transient protein expression in tobacco BY-2 plant cell packs using single and multi-cassette replicating vectors
Source: Plant Cell Rep. 2020 Apr 24;39(9):1115–27. doi: 10.1007/s00299-020-02544-w (PMC7223956; doi:10.1007/s00299-020-02544-w)
Supplement: Supplementary file 1 — Supplementary file1 (DOCX 447 kb) [file 299_2020_2544_MOESM1_ESM.docx]

**Supplementary Material**

**<Plant Cell Reports>**

**Transient protein expression in tobacco BY-2 plant cell packs using single and multi-cassette replicating vectors**

Zuzana Poborilova^1*^, Helena Plchova^1^, Noemi Cerovska^1^, Cornelius J. Gunter^2^, Inga I. Hitzeroth^2^, Edward P. Rybicki^2^, Tomas Moravec^1^

^1^ Institute of Experimental Botany of the Czech Academy of Sciences, Prague, Czech Republic

^2^ Biopharming Research Unit, Department of Molecular and Cell Biology, University of Cape Town, Cape Town, South Africa

*Corresponding author: Zuzana Poborilova

E-mail address: [poborilova@ueb.cas.cz](mailto:poborilova@ueb.cas.cz)

**Fig. S1** Fluorescence imaging in *Agrobacterium* suspensions

**Fig. S2** Western blot analysis of GFP and DsRed from *Agrobacterium* cells

**List S1** Sequences of multi-cassette pGB-R expression vectors

**Fig. S1** Fluorescence imaging in *Agrobacterium* suspensions. No GFP (Fig. S1a) and DsRed (Fig. S1b) fluorescence was noticed in *Agrobacterium* suspensions harbouring respective vectors compared to control *Agrobacterium* suspension when illuminated by UV and green light and observed under the yellow and red emission filter, respectively. The suspensions grown for 48 h ((OD)_600_ of 1.0-1.2) were subjected to the fluorescence imaging


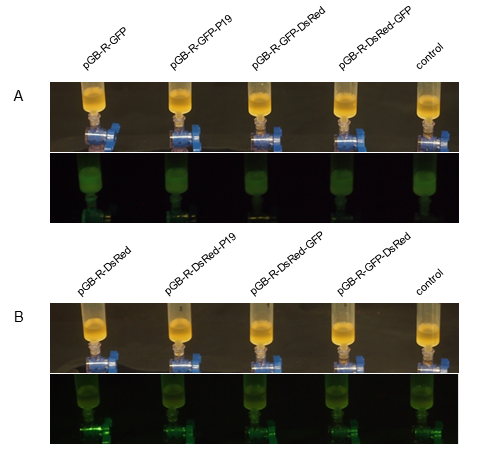


**Fig. S2** Western blot analysis of GFP and DsRed from *Agrobacterium* cells. The bands corresponding to 27-kDa of GFP (Fig. S2a) and 100-kDa of DsRed (tetramer) (Fig. S2b) were detected in the case of positive controls (wild type *Agrobacterium* + 100 ng of GFP and DsRed, respectively). No bands of 27-kDa in size corresponding to GFP and 28-kDa or 100-kDa corresponding to DsRed (monomer or tetramer) were determined in *Agrobacterium* cells harbouring studied vectors. Similarly, no bands were observed for negative control (wild type *Agrobacterium*)


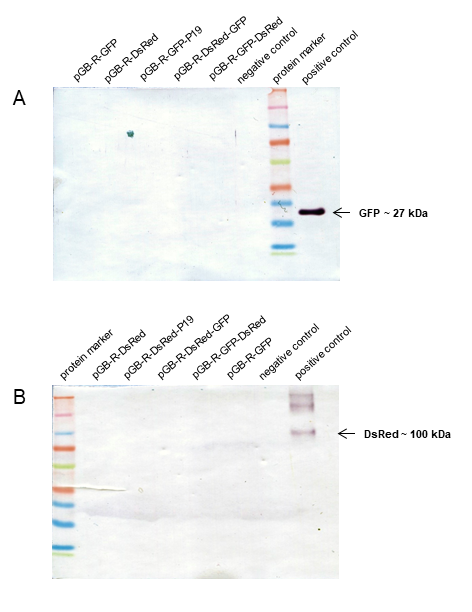


**List S1** Sequences of multi-cassette pGB-R expression vectors

**pGB-R-GFP**

Features:

pVS1 Sta: 1108 – 2108

pVS1-REP: 2701 – 3701

pBR322: 4111 – 4371

pBR322 ori: 4511 – 4791

Spm resistance: 5083 – 6093

Spm promoter: 6094 - 6227

Rb7-MAR: 6707-7874

SF: 7883-7917

LIR: 7930-8219

35S promoter: 8232 – 8546

5'UTR from CPMV RNA-2: 8547 - 9057

GFP: 9065 – 9784

3'UTR from CPMV RNA-2: 9827 – 10010

Nos terminator: 10064 – 10316

SIR: 10329 – 10481

Rep2A: 10486 – 10920

Rep: 10701 – 11576

LIR: 11578 – 11879

cgctgtcatgagaccggatcctgacaggatatattggcgggtaaacctaagagaaaagagcgtttattagaataatcgg

atatttaaaagggcgtgaaaaggtttatccgttcgtccatttgtatgtgcatgccaaccacagggttcccctcgggatc

aaagtactttgatccaacccctccgctgctatagtgcagtcggcttctgacgttcagtgcagccgtcatctgaaaacga

catgtcgcacaagtcctaagttacgcgacaggctgccgccctgcccttttcctggcgttttcttgtcgcgtgttttagt

cgcataaagtagaatacttgcgactagaaccggagacattacgccatgaacaagagcgccgccgctggcctgctgggct

atgcccgcgtcagcaccgacgaccaggacttgaccaaccaacgggccgaactgcacgcggccggctgcaccaagctgtt

ttccgagaagatcaccggcaccaggcgcgaccgcccggagctggccaggatgcttgaccacctacgccctggcgacgtt

gtgacagtgaccaggctagaccgcctggcccgcagcacccgcgacctactggacattgccgagcgcatccaggaggccg

gcgcgggcctgcgtagcctggcagagccgtgggccgacaccaccacgccggccggccgcatggtgttgaccgtgttcgc

cggcattgccgagttcgagcgttccctaatcatcgaccgcacccggagcgggcgcgaggccgccaaggcccgaggcgtg

aagtttggcccccgccctaccctcaccccggcacagatcgcgcacgcccgcgagctgatcgaccaggaaggccgcaccg

tgaaagaggcggctgcactgcttggcgtgcatcgctcgaccctgtaccgcgcacttgagcgcagcgaggaagtgacgcc

caccgaggccaggcggcgcggtgccttccgtgaggacgcattgaccgaggccgacgccctggcggccgccgagaatgaa

cgccaagaggaacaagcatgaaaccgcaccaggacggccaggacgaaccgtttttcattaccgaagagatcgaggcgga

gatgatcgcggccgggtacgtgttcgagccgcccgcgcacctctcaaccgtgcggctgcatgaaatcctggccggtttg

tctgatgccaagctggcggcctggccggccagcttggccgctgaagaaaccgagcgccgccgtctaaaaaggtgatgtg

tatttgagtaaaacagcttgcgtcatgcggtcgctgcgtatatgatccgatgagtaaataaacaaatacgcaaggggaa

cgcatgaaggttatcgctgtacttaaccagaaaggcgggtcaggcaagacgaccatcggaacccatctagcccgcgccc

tgcaactcgccggggccgatgttctgttagtcgattccgatccccagggcagtgcccgcgattgggcggccgtgcggga

agatcaaccgctaaccgttgtcggcatcgaccgcccgacgattgaccgcgacgtgaaggccatcggccggcgcgacttc

gtagtgatcgacggagcgccccaggcggcggacttggctgtgtccgcgatcaaggcagccgacttcgtgctgattccgg

tgcagccaagcccttacgacatatgggccaccgccgacctggtggagctggttaagcagcgcattgaggtcacggatgg

aaggctacaagcggcctttgtcgtgtcgcgggcgatcaaaggcacgcgcatcggcggtgaggttgccgaggcgctggcc

gggtacgagctgcccattcttgagtcccgtatcacgcagcgcgtgagctacccaggcactgccgccgccggcacaaccg

ttcttgaatcagaacccgagggcgacgctgcccgcgaggtccaggcgctggccgctgaaattaaatcaaaactcatttg

agttaatgaggtaaagagaaaatgagcaaaagcacaaacacgctaagtgccggccgtccgagcgcacgcagcagcaagg

ctgcaacgttggccagcctggcagacacgccagccatgaagcgggtcaactttcagttgccggcggaggatcacaccaa

gctgaagatgtacgcggtacgccaaggcaagaccattaccgagctgctatctgaatagatcgcgcagctaccagagtaa

atgagcaaatgaataaatgagtagatgaattttagcggctaaaggaggcggcatggaaaatcaagaacaaccaggcacc

gacgccgtggaatgccccatgtgtggaggaacgggcggttggccaggcgtaagcggctgggttgtctgccggccctgca

atggcactggaacccccaagcccgaggaatcggcgtgacggtcgcaaaccatccggcccggtacaaatcggcgcggcgc

tgggtgatgacctggtggagaagttgaaggccgcgcaggccgcccagcggcaacgcatcgaggcagaagcacgccccgg

tgaatcgtggcaagcggccgctgatcgaatccgcaaagaatcccggcaaccgccggcagccggtgcgccgtcgattagg

aagccgcccaagggcgacgagcaaccagattttttcgttccgatgctctatgacgtgggcacccgcgatagtcgcagca

tcatggacgtggccgttttccgtctgtcgaagcgtgaccgacgagctggcgaggtgatccgctacgagcttccagacgg

gcacgtagaggtttccgcagggccggccggcatggccagtgtgtgggattacgacctggtactgatggcggtttcccat

ctaaccgaatccatgaaccgataccgggaagggaagggagacaagcccggccgcgtgttccgtccacacgttgcggacg

tactcaagttctgccggcgagccgatggcggaaagcagaaagacgacctggtagaaacctgcattcggttaaacaccac

gcacgttgccatgcagcgtacgaagaaggccaagaacggccgcctggtgacggtatccgagggtgaagccttgattagc

cgctacaagatcgtaaagagcgaaaccgggcggccggagtacatcgagatcgagctagctgattggatgtaccgcgaga

tcacagaaggcaagaacccggacgtgctgacggttcaccccgattactttttgatcgatcccggcatcggccgttttct

ctaccgcctggcacgccgcgccgcaggcaaggcagaagccagatggttgttcaagacgatctacgaacgcagtggcagc

gccggagagttcaagaagttctgtttcaccgtgcgcaagctgatcgggtcaaatgacctgccggagtacgatttgaagg

aggaggcggggcaggctggcccgatcctagtcatgcgctaccgcaacctgatcgagggcgaagcatccgccggttccta

atgtacggagcagatgctagggcaaattgccctagcaggggaaaaaggtcgaaaaggactctttcctgtggatagcacg

tacattgggaacccaaagccgtacattgggaaccggaacccgtacattgggaacccaaagccgtacattgggaaccggt

cacacatgtaagtgactgatataaaagagaaaaaaggcgatttttccgcctaaaactctttaaaacttattaaaactct

taaaacccgcctggcctgtgcataactgtctggccagcgcacagccgaagagctgcaaaaagcgcctacccttcggtcg

ctgcgctccctacgccccgccgcttcgcgtcggcctatcgcggccgctggccgctcaaaaatggctggcctacggccag

gcaatctaccagggcgcggacaagccgcgccgtcgccactcgaccgccggcgcccacatcaaggcaccctgcctcgcgc

gtttcggtgatgacggtgaaaacctctgacacatgcagctcccggtgacggtcacagcttgtctgtaagcggatgccgg

gagcagacaagcccgtcagggcgcgtcagcgggtgttggcgggtgtcggggcgcagccatgacccagtcacgtagcgat

agcggagtgtatactggcttaactatgcggcatcagagcagattgtactgagagtgcaccatatgcggtgtgaaatacc

gcacagatgcgtaaggagaaaataccgcatcaggcgctcttccgcttcctcgctcactgactcgctgcgctcggtcgtt

cggctgcggcgagcggtatcagctcactcaaaggcggtaatacggttatccacagaatcaggggataacgcaggaaaga

acatgtgagcaaaaggccagcaaaaggccaggaaccgtaaaaaggccgcgttgctggcgtttttccataggctccgccc

ccctgacgagcatcacaaaaatcgacgctcaagtcagaggtggcgaaacccgacaggactataaagataccaggcgttt

ccccctggaagctccctcgtgcgctctcctgttccgaccctgccgcttaccggatacctgtccgcctttctcccttcgg

gaagcgtggcgctttctcatagctcacgctgtaggtatctcagttcggtgtaggtcgttcgctccaagctgggctgtgt

gcacgaaccccccgttcagcccgaccgctgcgccttatccggtaactatcgtcttgagtccaacccggtaagacacgac

ttatcgccactggcagcagccactggtaacaggattagcagagcgaggtatgtaggcggtgctacagagttcttgaagt

ggtggcctaactacggctacactagaaggacagtatttggtatctgcgctctgctgaagccagttaccttcggaaaaag

agttggtagctcttgatccggcaaacaaaccaccgctggtagcggtggtttttttgtttgcaagcagcagattacgcgc

agaaaaaaaggatctcaagaagatcctttgatcttttctacggggtctgacgctcagtggaacgaaaactcacgttaag

ggattttggtcatgcattctaggtgattatttgccgactaccttggtgatctcgcctttcacgtagtggacaaattctt

ccaactgatctgcgcgcgaggccaagcgatcttcttcttgtccaagataagcctgtctagcttcaagtatgacgggctg

atactgggccggcaggcgctccattgcccagtcggcagcgacatccttcggcgcgattttgccggttactgcgctgtac

caaatgcgggacaacgtaagcactacatttcgctcatcaccagcccagtcgggcggcgagttccatagcgttaaggttt

catttagcgcctcaaatagatcctgttcaggaaccggatcaaagagttcctccgccgctggacctaccaaggcaacgct

atgttctcttgcttttgtcagcaagatagccagatcaatgtcgatcgtggctggctcgaagatacctgcaagaatgtca

ttgcgctgccattctccaaattgcagttcgcgcttagctggataacgccacggaatgatgtcgtcgtgcacaacaatgg

tgacttctacagcgcggagaatctcgctctctccaggggaagccgaagtttccaaaaggtcgttgatcaaagctcgccg

cgttgtttcatcaagccttacggtcaccgtaaccagcaaatcaatatcactgtgtggcttcaggccgccatccactgcg

gagccgtacaaatgtacggccagcaacgtcggttcgagatggcgctcgatgacgccaactacctctgatagttgagtcg

atacttcggcgatcaccgcttccctcataatgtttaactttgttttagggcgactgccctgctgcgtaacatcgttgct

gctccataacatcaaacatcgacccacggcgtaacgcgcttgctgcttggatgcccgaggcatagactgtaccccaaaa

aaacagtcataacaagccatgaaaaccgccactgcgccgttaccaccgctgcgttcggtcaaggttctggaccagttgc

gtgagcgcatacgctacttgcattacagcttacgaaccgaacaggcttatgtccactgggttcgtgccttcatccgttt

ccacggtgtgcgtcacccggcaaccttgggtagcagcgaagtcgaggcatttctgtcctggctggaacagaacttatta

tttccttcctcttttctacagtatttaaagataccccaagaagctaattataacaagacgaactccaattcactgttcc

ttgcattctaaaaccttaaataccagaaaacagctttttcaaagttgttttcaaagttggcgtataacatagtatcgac

ggagccgattttgaaaccgcggtgatcacaggcagcaacgctctgtcatcgttacaatcaacatgctaccctccgcgag

atcatccgtgtttcaaacccggcagcttagttgccgttcttccgaatagcatcggtaacatgagcaaagtctgccgcct

tacaacggctctcccgctgacgccgtcccggactgatgggctgcctgtatcgagtggtgattttgtgccgagctgccgg

tcggggagctgttggctggctggtggcaggatatattgtggtgtaaacataacggatccggtctcaggagtcgattaaa

aatcccaattatatttggtctaatttagtttggtattgagtaaaacaaattcgaaccaaaccaaaatataaatatatag

tttttatatatatgcctttaagactttttatagaattttctttaaaaaatatctagaaatatttgcgactcttctggca

tgtaatatttcgttaaatatgaagtgctccatttttattaactttaaataattggttgtacgatcactttcttatcaag

tgttactaaaatgcgtcaatctctttgttcttccatattcatatgtcaaaatctatcaaaattcttatatatctttttc

gaatttgaagtgaaatttcgataatttaaaattaaatagaacatatcattatttaggtatcatattgatttttatactt

aattactaaatttggttaactttgaaagtgtacatcaacgaaaaattagtcaaacgactaaaataaataaatatcatgt

gttattaagaaaattctcctataagaatattttaatagatcatatgtttgtaaaaaaaattaatttttactaacacata

tatttacttatcaaaaatttgacaaagtaagattaaaataatattcatctaacaaaaaaaaaaccagaaaatgctgaaa

acccggcaaaaccgaaccaatccaaaccgatatagttggtttggtttgattttgatataaaccgaaccaactcggtcca

tttgcacccctaatcataatagctttaatatttcaagatattattaagttaacgttgtcaatatcctggaaattttgca

aaatgaatcaagcctatatggctgtaatatgaatttaaaagcagctcgatgtggtggtaatatgtaatttacttgattc

taaaaaaatatcccaagtattaataatttctgctaggaagaaggttagctacgatttacagcaaagccagaatacaaag

aaccataaagtgattgaagctcgaaatatacgaaggaacaaatatttttaaaaaaatacgcaatgacttggaacaaaag

aaagtgatatattttttgttcttaaacaagcatcccctctaaagaatggcagttttcctttgcatgtaactattatgct

cccttcgttacaaaaattttggactactattgggaacttcttctgaaaatagtcgctccatggagccctcaattgtact

accatcatttcttgttccgctgcttggaggtgactccgaggggttgcctcaaactctatcttataaccggcgtggaggc

atggaggcaggggtattttggtcattttaatagatagtggaaaatgacgtggaatttacttaaagacgaagtctttgcg

acaagggggggcccacgccgaatttaatattaccggcgtggcccccccttatcgcgagtgctttagcacgagcggtcca

gatttaaagtagaaaatttcccgcccactagggttaaaggtgttcacactataaaagcatatacgatgtgatggtattt

gctcgctggtaggagggaaacctcctcggattccattgcccagctatctgtcactttattgagaagatagtggaaaagg

aaggtggctcctacaaatgccatcattgcgataaaggaaaggccatcgttgaagatgcctctgccgacagtggtcccaa

agatggacccccacccacgaggagcatcgtggaaaaagaagacgttccaaccacgtcttcaaagcaagtggattgatgt

gatatctccactgacgtaagggatgacgcacaatcccactatccttcgcaagacccttcctctatataaggaagttcat

ttcatttggagaggtattaaaatcttaataggttttgataaaagcgaacgtggggaaacccgaaccaaaccttcttcta

aactctctctcatctctcttaaagcaaacttctctcttgtctttcttgcgtgagcgatcttcaacgttgtcagatcgtg

cttcggcaccagtacaacgttttctttcactgaagcgaaatcaaagatctctttgtggacacgtagtgcggcgccatta

aataacgtgtacttgtcctattcttgtcggtgtggtcttgggaaaagaaagcttgctggaggctgctgttcagccccat

acattacttgttacgattctgctgactttcggcgggtgcaatatctctacttctgcttgacgaggtattgttgcctgta

cttctttcttcttcttcttgctgattggttctataagaaatctagtattttctttgaaacagagttttcccgtggtttt

cgaacttggagaaagattgttaagcttctgtatattctgcccaaattcgcgaccggtaatggtgagcaagggcgaggag

ctgttcaccggggtggtgcccatcctggtcgagctggacggcgacgtaaacggccacaagttcagcgtgtccggcgagg

gcgagggcgatgccacctacggcaagctgaccctgaagttcatctgcaccaccggcaagctgcccgtgccctggcccac

cctcgtgaccaccttcagctacggcgtgcagtgcttcagccgctaccccgaccacatgaagcagcacgacttcttcaag

tccgccatgcccgaaggctacgtccaggagcgcaccatcttcttcaaggacgacggcaactacaagacccgcgccgagg

tgaagttcgagggcgacaccctggtgaaccgcatcgagctgaagggcatcgacttcaaggaggacggcaacatcctggg

gcacaagctggagtacaactacaacagccacaacgtctatatcatggccgacaagcagaagaacggcatcaaggtgaac

ttcaagatccgccacaacatcgaggacggcagcgtgcagctcgccgaccactaccagcagaacacccccatcggcgacg

gccccgtgctgctgcccgacaaccactacctgagcacccagtccgccctgagcaaagaccccaacgagaagcgcgatca

catggtcctgctggagttcgtgaccgccgccgggatcactcacggcatggacgagctgtacaagtaagctttcccgggc

atcaccatcaccatcactagctcgaggcctttaactctggtttcattaaattttctttagtttgaatttactgttattc

ggtgtgcatttctatgtttggtgagcggttttctgtgctcagagtgtgtttattttatgtaatttaatttctttgtgag

ctcctgtttagcaggtcgtcccttcagcaaggacacaaaaagattttaattttattaaaaaaaaaaaaaaaaaagaccg

ggaattcgatatcaagcttatcgacctgcagatcgttcaaacatttggcaataaagtttcttaagattgaatcctgttg

ccggtcttgcggtgattatcatataatttctgttgaattacgttaagcatgtaataattaacatgtaatgcatgacgtt

atttatgagatgggtttttatgattagagtcccgcaattatacatttaatacgcgatagaaaacaaaatatagcgcgca

aactaggataaattatcgcgcgcggtgtcatctatgttactagatccgctgtcaagcgaatgattattttatgaatata

tttcattgtgcaagtagatagaaattacatatgttacataacacacgaaataaacaaaaaaagacaatccaaaaacaaa

caccccaaaaaaaataatcactttagataaactcgtatgaggagaggcacgttaagctcagtgactcgacgattcccga

gcaaaaaaagtctccccgtcacacatatagtgggtgacgcaattatctttaaagtaatccttctgttgacttgtcattg

ataacatccagtcttcgtcaggattgcaaagaattatagaagggatcccaccttttattttcttcttttttccatattt

agggttgacagtgaaatcagactggcaacctattaattgcttccacaatgggacgaacttgaaggggatgtcgtcgatg

atattataggtggcgtgttcatcgtagttggtgaaatcgatggtaccgttccaatagttgtgtcgtccgagacttctag

cccaggtggtctttccggtacgagttggtccgcagatgtagaggctggggtgtcggattccattccttccattgtcctg

gttaaatcggccatccattcaaggtcagattgagcttgttggtatgagacaggatgtatgtaagtataagcgtctatgc

ttacatggtatagatgggtttccctccaggagtgtagatcttcgtggcagcgaagatctgattctgtgaagggcgacac

atacggttcaggttgtggagggaataatttgttggctgaatattccagccattgaagttttgttgcccattcatgaggg

aattcttccttgatcatgtcaagatattcctccttagacgttgcagtctggataatagttctccatcgtgcgtcagatt

tgcgaggagataccttatgatctcggaaatctcctctggttttaatatctccgtcctttgatatgtaatcaaggacttg

tttagagtttctagctggctggatattagggtgatttccttcaaaatcgaaaaaagaaggatccctaatacaaggtttt

ttatcaagctggagaagagcatgatagtgggtagtgccatcttgatgaagctcagaagcaacaccaaggaagaaaataa

gaaaaggtgtgagtttctcccagagaaactggaataaatcatctctttgagatgagcacttgggataggtaaggaaaac

atatttagattggagtctgaagttcttactagcagaaggcattttgttgtgactccgaggggttgcctcaaactctatc

ttataaccggcgtggaggcatggaggcaggggtattttggtcattttaatagatagtggaaaatgacgtggaatttact

taaagacgaagtctttgcgacaagggggggcccacgccgaatttaatattaccggcgtggcccccccttatcgcgagtg

ctttagcacgagcggtccagatttaaagtagaaaatttcccgcccactagggttaaaggtgttcacactatacaagcat

atacgatgtgatggtattgactagagtttctccgct

**pGB-R-DsRed**

Features:

pVS1 Sta: 1108 – 2108

pVS1-REP: 2701 – 3701

pBR322: 4111 – 4371

pBR322 ori: 4511 – 4791

Spm resistance: 5083 – 6093

Spm promoter: 6094 - 6227

Rb7-MAR: 6707-7874

SF: 7883-7917

LIR: 7930-8219

35S promoter: 8232 – 8546

5'UTR from CPMV RNA-2: 8547 - 9057

DsRed: 9065 – 9742

3'UTR from CPMV RNA-2: 9785 - 9968

Nos terminator: 10022 - 10274

SIR: 10287 - 10439

Rep2A: 10444 - 10878

Rep: 10659 - 11534

LIR: 11536 - 11837

cgctgtcatgagaccggatcctgacaggatatattggcgggtaaacctaagagaaaagagcgtttattagaataatcgg

atatttaaaagggcgtgaaaaggtttatccgttcgtccatttgtatgtgcatgccaaccacagggttcccctcgggatc

aaagtactttgatccaacccctccgctgctatagtgcagtcggcttctgacgttcagtgcagccgtcatctgaaaacga

catgtcgcacaagtcctaagttacgcgacaggctgccgccctgcccttttcctggcgttttcttgtcgcgtgttttagt

cgcataaagtagaatacttgcgactagaaccggagacattacgccatgaacaagagcgccgccgctggcctgctgggct

atgcccgcgtcagcaccgacgaccaggacttgaccaaccaacgggccgaactgcacgcggccggctgcaccaagctgtt

ttccgagaagatcaccggcaccaggcgcgaccgcccggagctggccaggatgcttgaccacctacgccctggcgacgtt

gtgacagtgaccaggctagaccgcctggcccgcagcacccgcgacctactggacattgccgagcgcatccaggaggccg

gcgcgggcctgcgtagcctggcagagccgtgggccgacaccaccacgccggccggccgcatggtgttgaccgtgttcgc

cggcattgccgagttcgagcgttccctaatcatcgaccgcacccggagcgggcgcgaggccgccaaggcccgaggcgtg

aagtttggcccccgccctaccctcaccccggcacagatcgcgcacgcccgcgagctgatcgaccaggaaggccgcaccg

tgaaagaggcggctgcactgcttggcgtgcatcgctcgaccctgtaccgcgcacttgagcgcagcgaggaagtgacgcc

caccgaggccaggcggcgcggtgccttccgtgaggacgcattgaccgaggccgacgccctggcggccgccgagaatgaa

cgccaagaggaacaagcatgaaaccgcaccaggacggccaggacgaaccgtttttcattaccgaagagatcgaggcgga

gatgatcgcggccgggtacgtgttcgagccgcccgcgcacctctcaaccgtgcggctgcatgaaatcctggccggtttg

tctgatgccaagctggcggcctggccggccagcttggccgctgaagaaaccgagcgccgccgtctaaaaaggtgatgtg

tatttgagtaaaacagcttgcgtcatgcggtcgctgcgtatatgatccgatgagtaaataaacaaatacgcaaggggaa

cgcatgaaggttatcgctgtacttaaccagaaaggcgggtcaggcaagacgaccatcggaacccatctagcccgcgccc

tgcaactcgccggggccgatgttctgttagtcgattccgatccccagggcagtgcccgcgattgggcggccgtgcggga

agatcaaccgctaaccgttgtcggcatcgaccgcccgacgattgaccgcgacgtgaaggccatcggccggcgcgacttc

gtagtgatcgacggagcgccccaggcggcggacttggctgtgtccgcgatcaaggcagccgacttcgtgctgattccgg

tgcagccaagcccttacgacatatgggccaccgccgacctggtggagctggttaagcagcgcattgaggtcacggatgg

aaggctacaagcggcctttgtcgtgtcgcgggcgatcaaaggcacgcgcatcggcggtgaggttgccgaggcgctggcc

gggtacgagctgcccattcttgagtcccgtatcacgcagcgcgtgagctacccaggcactgccgccgccggcacaaccg

ttcttgaatcagaacccgagggcgacgctgcccgcgaggtccaggcgctggccgctgaaattaaatcaaaactcatttg

agttaatgaggtaaagagaaaatgagcaaaagcacaaacacgctaagtgccggccgtccgagcgcacgcagcagcaagg

ctgcaacgttggccagcctggcagacacgccagccatgaagcgggtcaactttcagttgccggcggaggatcacaccaa

gctgaagatgtacgcggtacgccaaggcaagaccattaccgagctgctatctgaatagatcgcgcagctaccagagtaa

atgagcaaatgaataaatgagtagatgaattttagcggctaaaggaggcggcatggaaaatcaagaacaaccaggcacc

gacgccgtggaatgccccatgtgtggaggaacgggcggttggccaggcgtaagcggctgggttgtctgccggccctgca

atggcactggaacccccaagcccgaggaatcggcgtgacggtcgcaaaccatccggcccggtacaaatcggcgcggcgc

tgggtgatgacctggtggagaagttgaaggccgcgcaggccgcccagcggcaacgcatcgaggcagaagcacgccccgg

tgaatcgtggcaagcggccgctgatcgaatccgcaaagaatcccggcaaccgccggcagccggtgcgccgtcgattagg

aagccgcccaagggcgacgagcaaccagattttttcgttccgatgctctatgacgtgggcacccgcgatagtcgcagca

tcatggacgtggccgttttccgtctgtcgaagcgtgaccgacgagctggcgaggtgatccgctacgagcttccagacgg

gcacgtagaggtttccgcagggccggccggcatggccagtgtgtgggattacgacctggtactgatggcggtttcccat

ctaaccgaatccatgaaccgataccgggaagggaagggagacaagcccggccgcgtgttccgtccacacgttgcggacg

tactcaagttctgccggcgagccgatggcggaaagcagaaagacgacctggtagaaacctgcattcggttaaacaccac

gcacgttgccatgcagcgtacgaagaaggccaagaacggccgcctggtgacggtatccgagggtgaagccttgattagc

cgctacaagatcgtaaagagcgaaaccgggcggccggagtacatcgagatcgagctagctgattggatgtaccgcgaga

tcacagaaggcaagaacccggacgtgctgacggttcaccccgattactttttgatcgatcccggcatcggccgttttct

ctaccgcctggcacgccgcgccgcaggcaaggcagaagccagatggttgttcaagacgatctacgaacgcagtggcagc

gccggagagttcaagaagttctgtttcaccgtgcgcaagctgatcgggtcaaatgacctgccggagtacgatttgaagg

aggaggcggggcaggctggcccgatcctagtcatgcgctaccgcaacctgatcgagggcgaagcatccgccggttccta

atgtacggagcagatgctagggcaaattgccctagcaggggaaaaaggtcgaaaaggactctttcctgtggatagcacg

tacattgggaacccaaagccgtacattgggaaccggaacccgtacattgggaacccaaagccgtacattgggaaccggt

cacacatgtaagtgactgatataaaagagaaaaaaggcgatttttccgcctaaaactctttaaaacttattaaaactct

taaaacccgcctggcctgtgcataactgtctggccagcgcacagccgaagagctgcaaaaagcgcctacccttcggtcg

ctgcgctccctacgccccgccgcttcgcgtcggcctatcgcggccgctggccgctcaaaaatggctggcctacggccag

gcaatctaccagggcgcggacaagccgcgccgtcgccactcgaccgccggcgcccacatcaaggcaccctgcctcgcgc

gtttcggtgatgacggtgaaaacctctgacacatgcagctcccggtgacggtcacagcttgtctgtaagcggatgccgg

gagcagacaagcccgtcagggcgcgtcagcgggtgttggcgggtgtcggggcgcagccatgacccagtcacgtagcgat

agcggagtgtatactggcttaactatgcggcatcagagcagattgtactgagagtgcaccatatgcggtgtgaaatacc

gcacagatgcgtaaggagaaaataccgcatcaggcgctcttccgcttcctcgctcactgactcgctgcgctcggtcgtt

cggctgcggcgagcggtatcagctcactcaaaggcggtaatacggttatccacagaatcaggggataacgcaggaaaga

acatgtgagcaaaaggccagcaaaaggccaggaaccgtaaaaaggccgcgttgctggcgtttttccataggctccgccc

ccctgacgagcatcacaaaaatcgacgctcaagtcagaggtggcgaaacccgacaggactataaagataccaggcgttt

ccccctggaagctccctcgtgcgctctcctgttccgaccctgccgcttaccggatacctgtccgcctttctcccttcgg

gaagcgtggcgctttctcatagctcacgctgtaggtatctcagttcggtgtaggtcgttcgctccaagctgggctgtgt

gcacgaaccccccgttcagcccgaccgctgcgccttatccggtaactatcgtcttgagtccaacccggtaagacacgac

ttatcgccactggcagcagccactggtaacaggattagcagagcgaggtatgtaggcggtgctacagagttcttgaagt

ggtggcctaactacggctacactagaaggacagtatttggtatctgcgctctgctgaagccagttaccttcggaaaaag

agttggtagctcttgatccggcaaacaaaccaccgctggtagcggtggtttttttgtttgcaagcagcagattacgcgc

agaaaaaaaggatctcaagaagatcctttgatcttttctacggggtctgacgctcagtggaacgaaaactcacgttaag

ggattttggtcatgcattctaggtgattatttgccgactaccttggtgatctcgcctttcacgtagtggacaaattctt

ccaactgatctgcgcgcgaggccaagcgatcttcttcttgtccaagataagcctgtctagcttcaagtatgacgggctg

atactgggccggcaggcgctccattgcccagtcggcagcgacatccttcggcgcgattttgccggttactgcgctgtac

caaatgcgggacaacgtaagcactacatttcgctcatcaccagcccagtcgggcggcgagttccatagcgttaaggttt

catttagcgcctcaaatagatcctgttcaggaaccggatcaaagagttcctccgccgctggacctaccaaggcaacgct

atgttctcttgcttttgtcagcaagatagccagatcaatgtcgatcgtggctggctcgaagatacctgcaagaatgtca

ttgcgctgccattctccaaattgcagttcgcgcttagctggataacgccacggaatgatgtcgtcgtgcacaacaatgg

tgacttctacagcgcggagaatctcgctctctccaggggaagccgaagtttccaaaaggtcgttgatcaaagctcgccg

cgttgtttcatcaagccttacggtcaccgtaaccagcaaatcaatatcactgtgtggcttcaggccgccatccactgcg

gagccgtacaaatgtacggccagcaacgtcggttcgagatggcgctcgatgacgccaactacctctgatagttgagtcg

atacttcggcgatcaccgcttccctcataatgtttaactttgttttagggcgactgccctgctgcgtaacatcgttgct

gctccataacatcaaacatcgacccacggcgtaacgcgcttgctgcttggatgcccgaggcatagactgtaccccaaaa

aaacagtcataacaagccatgaaaaccgccactgcgccgttaccaccgctgcgttcggtcaaggttctggaccagttgc

gtgagcgcatacgctacttgcattacagcttacgaaccgaacaggcttatgtccactgggttcgtgccttcatccgttt

ccacggtgtgcgtcacccggcaaccttgggtagcagcgaagtcgaggcatttctgtcctggctggaacagaacttatta

tttccttcctcttttctacagtatttaaagataccccaagaagctaattataacaagacgaactccaattcactgttcc

ttgcattctaaaaccttaaataccagaaaacagctttttcaaagttgttttcaaagttggcgtataacatagtatcgac

ggagccgattttgaaaccgcggtgatcacaggcagcaacgctctgtcatcgttacaatcaacatgctaccctccgcgag

atcatccgtgtttcaaacccggcagcttagttgccgttcttccgaatagcatcggtaacatgagcaaagtctgccgcct

tacaacggctctcccgctgacgccgtcccggactgatgggctgcctgtatcgagtggtgattttgtgccgagctgccgg

tcggggagctgttggctggctggtggcaggatatattgtggtgtaaacataacggatccggtctcaggagtcgattaaa

aatcccaattatatttggtctaatttagtttggtattgagtaaaacaaattcgaaccaaaccaaaatataaatatatag

tttttatatatatgcctttaagactttttatagaattttctttaaaaaatatctagaaatatttgcgactcttctggca

tgtaatatttcgttaaatatgaagtgctccatttttattaactttaaataattggttgtacgatcactttcttatcaag

tgttactaaaatgcgtcaatctctttgttcttccatattcatatgtcaaaatctatcaaaattcttatatatctttttc

gaatttgaagtgaaatttcgataatttaaaattaaatagaacatatcattatttaggtatcatattgatttttatactt

aattactaaatttggttaactttgaaagtgtacatcaacgaaaaattagtcaaacgactaaaataaataaatatcatgt

gttattaagaaaattctcctataagaatattttaatagatcatatgtttgtaaaaaaaattaatttttactaacacata

tatttacttatcaaaaatttgacaaagtaagattaaaataatattcatctaacaaaaaaaaaaccagaaaatgctgaaa

acccggcaaaaccgaaccaatccaaaccgatatagttggtttggtttgattttgatataaaccgaaccaactcggtcca

tttgcacccctaatcataatagctttaatatttcaagatattattaagttaacgttgtcaatatcctggaaattttgca

aaatgaatcaagcctatatggctgtaatatgaatttaaaagcagctcgatgtggtggtaatatgtaatttacttgattc

taaaaaaatatcccaagtattaataatttctgctaggaagaaggttagctacgatttacagcaaagccagaatacaaag

aaccataaagtgattgaagctcgaaatatacgaaggaacaaatatttttaaaaaaatacgcaatgacttggaacaaaag

aaagtgatatattttttgttcttaaacaagcatcccctctaaagaatggcagttttcctttgcatgtaactattatgct

cccttcgttacaaaaattttggactactattgggaacttcttctgaaaatagtcgctccatggagccctcaattgtact

accatcatttcttgttccgctgcttggaggtgactccgaggggttgcctcaaactctatcttataaccggcgtggaggc

atggaggcaggggtattttggtcattttaatagatagtggaaaatgacgtggaatttacttaaagacgaagtctttgcg

acaagggggggcccacgccgaatttaatattaccggcgtggcccccccttatcgcgagtgctttagcacgagcggtcca

gatttaaagtagaaaatttcccgcccactagggttaaaggtgttcacactataaaagcatatacgatgtgatggtattt

gctcgctggtaggagggaaacctcctcggattccattgcccagctatctgtcactttattgagaagatagtggaaaagg

aaggtggctcctacaaatgccatcattgcgataaaggaaaggccatcgttgaagatgcctctgccgacagtggtcccaa

agatggacccccacccacgaggagcatcgtggaaaaagaagacgttccaaccacgtcttcaaagcaagtggattgatgt

gatatctccactgacgtaagggatgacgcacaatcccactatccttcgcaagacccttcctctatataaggaagttcat

ttcatttggagaggtattaaaatcttaataggttttgataaaagcgaacgtggggaaacccgaaccaaaccttcttcta

aactctctctcatctctcttaaagcaaacttctctcttgtctttcttgcgtgagcgatcttcaacgttgtcagatcgtg

cttcggcaccagtacaacgttttctttcactgaagcgaaatcaaagatctctttgtggacacgtagtgcggcgccatta

aataacgtgtacttgtcctattcttgtcggtgtggtcttgggaaaagaaagcttgctggaggctgctgttcagccccat

acattacttgttacgattctgctgactttcggcgggtgcaatatctctacttctgcttgacgaggtattgttgcctgta

cttctttcttcttcttcttgctgattggttctataagaaatctagtattttctttgaaacagagttttcccgtggtttt

cgaacttggagaaagattgttaagcttctgtatattctgcccaaattcgcgaccggtaatggggtcatccaagaatgtt

atcaaggagttcatgaggtttaaggttcgcatggaaggaacggtcaatgggcacgagtttgaaatagaaggcgaaggag

aggggaggccatacgaaggccacaataccgtaaagcttaaggtaaccaaggggggacctttgccatttgcttgggatat

tttgtcaccacaatttcagtatggaagcaaggtatatgtcaagcaccctgccgacataccagactataaaaagctgtca

tttcctgaaggatttaaatgggaaagggtcatgaactttgaagatggtggcgtcgttactgtaacccaggattccagtt

tgcaggatggctgtttcatctacaaggtcaagttcattggcgtgaactttccttccgatggacctgttatgcaaaagaa

aacaatgggctgggaagccagcactgagcgtttgtatcctcgtgatggcgtgttgaaaggagagattcataaggctctg

aagctgaaagacggtggtcattacctagttgaattcaaaagtatttacatggcaaagaagcctgtgcagctaccagggt

actactatgttgactccaaactggatataacaagccacaacgaagattatacaatcgttgagcagtatgaaagaaccga

gggacgccaccatctgttcctttaagctttcccgggcatcaccatcaccatcactagctcgaggcctttaactctggtt

tcattaaattttctttagtttgaatttactgttattcggtgtgcatttctatgtttggtgagcggttttctgtgctcag

agtgtgtttattttatgtaatttaatttctttgtgagctcctgtttagcaggtcgtcccttcagcaaggacacaaaaag

attttaattttattaaaaaaaaaaaaaaaaaagaccgggaattcgatatcaagcttatcgacctgcagatcgttcaaac

atttggcaataaagtttcttaagattgaatcctgttgccggtcttgcggtgattatcatataatttctgttgaattacg

ttaagcatgtaataattaacatgtaatgcatgacgttatttatgagatgggtttttatgattagagtcccgcaattata

catttaatacgcgatagaaaacaaaatatagcgcgcaaactaggataaattatcgcgcgcggtgtcatctatgttacta

gatccgctgtcaagcgaatgattattttatgaatatatttcattgtgcaagtagatagaaattacatatgttacataac

acacgaaataaacaaaaaaagacaatccaaaaacaaacaccccaaaaaaaataatcactttagataaactcgtatgagg

agaggcacgttaagctcagtgactcgacgattcccgagcaaaaaaagtctccccgtcacacatatagtgggtgacgcaa

ttatctttaaagtaatccttctgttgacttgtcattgataacatccagtcttcgtcaggattgcaaagaattatagaag

ggatcccaccttttattttcttcttttttccatatttagggttgacagtgaaatcagactggcaacctattaattgctt

ccacaatgggacgaacttgaaggggatgtcgtcgatgatattataggtggcgtgttcatcgtagttggtgaaatcgatg

gtaccgttccaatagttgtgtcgtccgagacttctagcccaggtggtctttccggtacgagttggtccgcagatgtaga

ggctggggtgtcggattccattccttccattgtcctggttaaatcggccatccattcaaggtcagattgagcttgttgg

tatgagacaggatgtatgtaagtataagcgtctatgcttacatggtatagatgggtttccctccaggagtgtagatctt

cgtggcagcgaagatctgattctgtgaagggcgacacatacggttcaggttgtggagggaataatttgttggctgaata

ttccagccattgaagttttgttgcccattcatgagggaattcttccttgatcatgtcaagatattcctccttagacgtt

gcagtctggataatagttctccatcgtgcgtcagatttgcgaggagataccttatgatctcggaaatctcctctggttt

taatatctccgtcctttgatatgtaatcaaggacttgtttagagtttctagctggctggatattagggtgatttccttc

aaaatcgaaaaaagaaggatccctaatacaaggttttttatcaagctggagaagagcatgatagtgggtagtgccatct

tgatgaagctcagaagcaacaccaaggaagaaaataagaaaaggtgtgagtttctcccagagaaactggaataaatcat

ctctttgagatgagcacttgggataggtaaggaaaacatatttagattggagtctgaagttcttactagcagaaggcat

tttgttgtgactccgaggggttgcctcaaactctatcttataaccggcgtggaggcatggaggcaggggtattttggtc

attttaatagatagtggaaaatgacgtggaatttacttaaagacgaagtctttgcgacaagggggggcccacgccgaat

ttaatattaccggcgtggcccccccttatcgcgagtgctttagcacgagcggtccagatttaaagtagaaaatttcccg

cccactagggttaaaggtgttcacactatacaagcatatacgatgtgatggtattgactagagtttctccgct

**pGB-R-GFP-P19**

Features:

pVS1 Sta: 1108 – 2108

pVS1-REP: 2701 – 3701

pBR322: 4111 – 4371

pBR322 ori: 4511 – 4791

Spm resistance: 5083 – 6093

Spm promoter: 6094 - 6227

35S promoter: 6711 – 7136

TMV omega: 7153 – 7214

P19: 7224 – 7742

Nos terminator: 7755 – 8007

SF: 8018 - 8052

LIR: 8065 - 8354

35S promoter: 8367 - 8681

5'UTR from CPMV RNA-2: 8682 - 9192

GFP: 9200 - 9919

3'UTR from CPMV RNA-2: 9962 - 10145

Nos terminator: 10199 - 10451

SIR: 10464 - 10616

Rep2A: 10621 - 11055

Rep: 10836 - 11711

LIR: 11713 - 12014

cgctgtcatgagaccggatcctgacaggatatattggcgggtaaacctaagagaaaagagcgtttattagaataatcgg

atatttaaaagggcgtgaaaaggtttatccgttcgtccatttgtatgtgcatgccaaccacagggttcccctcgggatc

aaagtactttgatccaacccctccgctgctatagtgcagtcggcttctgacgttcagtgcagccgtcatctgaaaacga

catgtcgcacaagtcctaagttacgcgacaggctgccgccctgcccttttcctggcgttttcttgtcgcgtgttttagt

cgcataaagtagaatacttgcgactagaaccggagacattacgccatgaacaagagcgccgccgctggcctgctgggct

atgcccgcgtcagcaccgacgaccaggacttgaccaaccaacgggccgaactgcacgcggccggctgcaccaagctgtt

ttccgagaagatcaccggcaccaggcgcgaccgcccggagctggccaggatgcttgaccacctacgccctggcgacgtt

gtgacagtgaccaggctagaccgcctggcccgcagcacccgcgacctactggacattgccgagcgcatccaggaggccg

gcgcgggcctgcgtagcctggcagagccgtgggccgacaccaccacgccggccggccgcatggtgttgaccgtgttcgc

cggcattgccgagttcgagcgttccctaatcatcgaccgcacccggagcgggcgcgaggccgccaaggcccgaggcgtg

aagtttggcccccgccctaccctcaccccggcacagatcgcgcacgcccgcgagctgatcgaccaggaaggccgcaccg

tgaaagaggcggctgcactgcttggcgtgcatcgctcgaccctgtaccgcgcacttgagcgcagcgaggaagtgacgcc

caccgaggccaggcggcgcggtgccttccgtgaggacgcattgaccgaggccgacgccctggcggccgccgagaatgaa

cgccaagaggaacaagcatgaaaccgcaccaggacggccaggacgaaccgtttttcattaccgaagagatcgaggcgga

gatgatcgcggccgggtacgtgttcgagccgcccgcgcacctctcaaccgtgcggctgcatgaaatcctggccggtttg

tctgatgccaagctggcggcctggccggccagcttggccgctgaagaaaccgagcgccgccgtctaaaaaggtgatgtg

tatttgagtaaaacagcttgcgtcatgcggtcgctgcgtatatgatccgatgagtaaataaacaaatacgcaaggggaa

cgcatgaaggttatcgctgtacttaaccagaaaggcgggtcaggcaagacgaccatcggaacccatctagcccgcgccc

tgcaactcgccggggccgatgttctgttagtcgattccgatccccagggcagtgcccgcgattgggcggccgtgcggga

agatcaaccgctaaccgttgtcggcatcgaccgcccgacgattgaccgcgacgtgaaggccatcggccggcgcgacttc

gtagtgatcgacggagcgccccaggcggcggacttggctgtgtccgcgatcaaggcagccgacttcgtgctgattccgg

tgcagccaagcccttacgacatatgggccaccgccgacctggtggagctggttaagcagcgcattgaggtcacggatgg

aaggctacaagcggcctttgtcgtgtcgcgggcgatcaaaggcacgcgcatcggcggtgaggttgccgaggcgctggcc

gggtacgagctgcccattcttgagtcccgtatcacgcagcgcgtgagctacccaggcactgccgccgccggcacaaccg

ttcttgaatcagaacccgagggcgacgctgcccgcgaggtccaggcgctggccgctgaaattaaatcaaaactcatttg

agttaatgaggtaaagagaaaatgagcaaaagcacaaacacgctaagtgccggccgtccgagcgcacgcagcagcaagg

ctgcaacgttggccagcctggcagacacgccagccatgaagcgggtcaactttcagttgccggcggaggatcacaccaa

gctgaagatgtacgcggtacgccaaggcaagaccattaccgagctgctatctgaatagatcgcgcagctaccagagtaa

atgagcaaatgaataaatgagtagatgaattttagcggctaaaggaggcggcatggaaaatcaagaacaaccaggcacc

gacgccgtggaatgccccatgtgtggaggaacgggcggttggccaggcgtaagcggctgggttgtctgccggccctgca

atggcactggaacccccaagcccgaggaatcggcgtgacggtcgcaaaccatccggcccggtacaaatcggcgcggcgc

tgggtgatgacctggtggagaagttgaaggccgcgcaggccgcccagcggcaacgcatcgaggcagaagcacgccccgg

tgaatcgtggcaagcggccgctgatcgaatccgcaaagaatcccggcaaccgccggcagccggtgcgccgtcgattagg

aagccgcccaagggcgacgagcaaccagattttttcgttccgatgctctatgacgtgggcacccgcgatagtcgcagca

tcatggacgtggccgttttccgtctgtcgaagcgtgaccgacgagctggcgaggtgatccgctacgagcttccagacgg

gcacgtagaggtttccgcagggccggccggcatggccagtgtgtgggattacgacctggtactgatggcggtttcccat

ctaaccgaatccatgaaccgataccgggaagggaagggagacaagcccggccgcgtgttccgtccacacgttgcggacg

tactcaagttctgccggcgagccgatggcggaaagcagaaagacgacctggtagaaacctgcattcggttaaacaccac

gcacgttgccatgcagcgtacgaagaaggccaagaacggccgcctggtgacggtatccgagggtgaagccttgattagc

cgctacaagatcgtaaagagcgaaaccgggcggccggagtacatcgagatcgagctagctgattggatgtaccgcgaga

tcacagaaggcaagaacccggacgtgctgacggttcaccccgattactttttgatcgatcccggcatcggccgttttct

ctaccgcctggcacgccgcgccgcaggcaaggcagaagccagatggttgttcaagacgatctacgaacgcagtggcagc

gccggagagttcaagaagttctgtttcaccgtgcgcaagctgatcgggtcaaatgacctgccggagtacgatttgaagg

aggaggcggggcaggctggcccgatcctagtcatgcgctaccgcaacctgatcgagggcgaagcatccgccggttccta

atgtacggagcagatgctagggcaaattgccctagcaggggaaaaaggtcgaaaaggactctttcctgtggatagcacg

tacattgggaacccaaagccgtacattgggaaccggaacccgtacattgggaacccaaagccgtacattgggaaccggt

cacacatgtaagtgactgatataaaagagaaaaaaggcgatttttccgcctaaaactctttaaaacttattaaaactct

taaaacccgcctggcctgtgcataactgtctggccagcgcacagccgaagagctgcaaaaagcgcctacccttcggtcg

ctgcgctccctacgccccgccgcttcgcgtcggcctatcgcggccgctggccgctcaaaaatggctggcctacggccag

gcaatctaccagggcgcggacaagccgcgccgtcgccactcgaccgccggcgcccacatcaaggcaccctgcctcgcgc

gtttcggtgatgacggtgaaaacctctgacacatgcagctcccggtgacggtcacagcttgtctgtaagcggatgccgg

gagcagacaagcccgtcagggcgcgtcagcgggtgttggcgggtgtcggggcgcagccatgacccagtcacgtagcgat

agcggagtgtatactggcttaactatgcggcatcagagcagattgtactgagagtgcaccatatgcggtgtgaaatacc

gcacagatgcgtaaggagaaaataccgcatcaggcgctcttccgcttcctcgctcactgactcgctgcgctcggtcgtt

cggctgcggcgagcggtatcagctcactcaaaggcggtaatacggttatccacagaatcaggggataacgcaggaaaga

acatgtgagcaaaaggccagcaaaaggccaggaaccgtaaaaaggccgcgttgctggcgtttttccataggctccgccc

ccctgacgagcatcacaaaaatcgacgctcaagtcagaggtggcgaaacccgacaggactataaagataccaggcgttt

ccccctggaagctccctcgtgcgctctcctgttccgaccctgccgcttaccggatacctgtccgcctttctcccttcgg

gaagcgtggcgctttctcatagctcacgctgtaggtatctcagttcggtgtaggtcgttcgctccaagctgggctgtgt

gcacgaaccccccgttcagcccgaccgctgcgccttatccggtaactatcgtcttgagtccaacccggtaagacacgac

ttatcgccactggcagcagccactggtaacaggattagcagagcgaggtatgtaggcggtgctacagagttcttgaagt

ggtggcctaactacggctacactagaaggacagtatttggtatctgcgctctgctgaagccagttaccttcggaaaaag

agttggtagctcttgatccggcaaacaaaccaccgctggtagcggtggtttttttgtttgcaagcagcagattacgcgc

agaaaaaaaggatctcaagaagatcctttgatcttttctacggggtctgacgctcagtggaacgaaaactcacgttaag

ggattttggtcatgcattctaggtgattatttgccgactaccttggtgatctcgcctttcacgtagtggacaaattctt

ccaactgatctgcgcgcgaggccaagcgatcttcttcttgtccaagataagcctgtctagcttcaagtatgacgggctg

atactgggccggcaggcgctccattgcccagtcggcagcgacatccttcggcgcgattttgccggttactgcgctgtac

caaatgcgggacaacgtaagcactacatttcgctcatcaccagcccagtcgggcggcgagttccatagcgttaaggttt

catttagcgcctcaaatagatcctgttcaggaaccggatcaaagagttcctccgccgctggacctaccaaggcaacgct

atgttctcttgcttttgtcagcaagatagccagatcaatgtcgatcgtggctggctcgaagatacctgcaagaatgtca

ttgcgctgccattctccaaattgcagttcgcgcttagctggataacgccacggaatgatgtcgtcgtgcacaacaatgg

tgacttctacagcgcggagaatctcgctctctccaggggaagccgaagtttccaaaaggtcgttgatcaaagctcgccg

cgttgtttcatcaagccttacggtcaccgtaaccagcaaatcaatatcactgtgtggcttcaggccgccatccactgcg

gagccgtacaaatgtacggccagcaacgtcggttcgagatggcgctcgatgacgccaactacctctgatagttgagtcg

atacttcggcgatcaccgcttccctcataatgtttaactttgttttagggcgactgccctgctgcgtaacatcgttgct

gctccataacatcaaacatcgacccacggcgtaacgcgcttgctgcttggatgcccgaggcatagactgtaccccaaaa

aaacagtcataacaagccatgaaaaccgccactgcgccgttaccaccgctgcgttcggtcaaggttctggaccagttgc

gtgagcgcatacgctacttgcattacagcttacgaaccgaacaggcttatgtccactgggttcgtgccttcatccgttt

ccacggtgtgcgtcacccggcaaccttgggtagcagcgaagtcgaggcatttctgtcctggctggaacagaacttatta

tttccttcctcttttctacagtatttaaagataccccaagaagctaattataacaagacgaactccaattcactgttcc

ttgcattctaaaaccttaaataccagaaaacagctttttcaaagttgttttcaaagttggcgtataacatagtatcgac

ggagccgattttgaaaccgcggtgatcacaggcagcaacgctctgtcatcgttacaatcaacatgctaccctccgcgag

atcatccgtgtttcaaacccggcagcttagttgccgttcttccgaatagcatcggtaacatgagcaaagtctgccgcct

tacaacggctctcccgctgacgccgtcccggactgatgggctgcctgtatcgagtggtgattttgtgccgagctgccgg

tcggggagctgttggctggctggtggcaggatatattgtggtgtaaacataacggatccggtctcaggagggaggtcaa

catggtggagcacgacactctggtctactccaaaaatgtcaaagatacagtctcagaagatcaaagggctattgagact

tttcaacaaaggataatttcgggaaacctcctcggattccattgcccagctatctgtcacttcatcgaaaggacagtag

aaaaggaaggtggctcctacaaatgccatcattgcgataaaggaaaggctatcattcaagatctctctgccgacagtgg

tcccaaagatggacccccacccacgaggagcatcgtggaaaaagaagaggttccaaccacgtctacaaagcaagtggat

tgatgtgacatctccactgacgtaagggatgacgcacaatcccactatccttcgcaagacccttcctctatataaggaa

gttcatttcatttggagaggacacgctcgagtataagagctcatttttacaacaattaccaacaacaacaaacaacaaa

caacattacaattacatttacaattatcgatacaatggaacgagctatacaaggaaacgacgctagggaacaagctaac

agtgaacgttgggatggaggatcaggaggtaccacttctcccttcaaacttcctgacgaaagtccgagttggactgagt

ggcggctacataacgatgagactaattcgaatcaagataatccccttggtttcaaggaaagctggggtttcgggaaagt

tgtatttaagagatatctcagatacgacaggacggaagcttcactgcacagagtccttggatcttggacgggagattcg

gttaactatgcagcatctcgatttttcggtttcgaccagatcggatgtacctatagtattcggtttcgaggagttagta

tcaccgtttctggaggctctcgaactcttcagcatctctgtgagatggcaattcggtctaagcaagaactgctacagct

tgccccaatcgaagtggaaagtaatgtatcaagaggatgccctgaaggtactgaaaccttcgaaaaagaaagcgagtga

gcttgtcaagcagatcgttcaaacatttggcaataaagtttcttaagattgaatcctgttgccggtcttgcgatgatta

tcatataatttctgttgaattacgttaagcatgtaataattaacatgtaatgcatgacgttatttatgagatgggtttt

tatgattagagtcccgcaattatacatttaatacgcgatagaaaacaaaatatagcgcgcaaactaggataaattatcg

cgcgcggtgtcatctatgttactagatcgacgctccatggagccctcaattgtactaccatcatttcttgttccgctgc

ttggaggtgactccgaggggttgcctcaaactctatcttataaccggcgtggaggcatggaggcaggggtattttggtc

attttaatagatagtggaaaatgacgtggaatttacttaaagacgaagtctttgcgacaagggggggcccacgccgaat

ttaatattaccggcgtggcccccccttatcgcgagtgctttagcacgagcggtccagatttaaagtagaaaatttcccg

cccactagggttaaaggtgttcacactataaaagcatatacgatgtgatggtatttgctcgctggtaggagggaaacct

cctcggattccattgcccagctatctgtcactttattgagaagatagtggaaaaggaaggtggctcctacaaatgccat

cattgcgataaaggaaaggccatcgttgaagatgcctctgccgacagtggtcccaaagatggacccccacccacgagga

gcatcgtggaaaaagaagacgttccaaccacgtcttcaaagcaagtggattgatgtgatatctccactgacgtaaggga

tgacgcacaatcccactatccttcgcaagacccttcctctatataaggaagttcatttcatttggagaggtattaaaat

cttaataggttttgataaaagcgaacgtggggaaacccgaaccaaaccttcttctaaactctctctcatctctcttaaa

gcaaacttctctcttgtctttcttgcgtgagcgatcttcaacgttgtcagatcgtgcttcggcaccagtacaacgtttt

ctttcactgaagcgaaatcaaagatctctttgtggacacgtagtgcggcgccattaaataacgtgtacttgtcctattc

ttgtcggtgtggtcttgggaaaagaaagcttgctggaggctgctgttcagccccatacattacttgttacgattctgct

gactttcggcgggtgcaatatctctacttctgcttgacgaggtattgttgcctgtacttctttcttcttcttcttgctg

attggttctataagaaatctagtattttctttgaaacagagttttcccgtggttttcgaacttggagaaagattgttaa

gcttctgtatattctgcccaaattcgcgaccggtaatggtgagcaagggcgaggagctgttcaccggggtggtgcccat

cctggtcgagctggacggcgacgtaaacggccacaagttcagcgtgtccggcgagggcgagggcgatgccacctacggc

aagctgaccctgaagttcatctgcaccaccggcaagctgcccgtgccctggcccaccctcgtgaccaccttcagctacg

gcgtgcagtgcttcagccgctaccccgaccacatgaagcagcacgacttcttcaagtccgccatgcccgaaggctacgt

ccaggagcgcaccatcttcttcaaggacgacggcaactacaagacccgcgccgaggtgaagttcgagggcgacaccctg

gtgaaccgcatcgagctgaagggcatcgacttcaaggaggacggcaacatcctggggcacaagctggagtacaactaca

acagccacaacgtctatatcatggccgacaagcagaagaacggcatcaaggtgaacttcaagatccgccacaacatcga

ggacggcagcgtgcagctcgccgaccactaccagcagaacacccccatcggcgacggccccgtgctgctgcccgacaac

cactacctgagcacccagtccgccctgagcaaagaccccaacgagaagcgcgatcacatggtcctgctggagttcgtga

ccgccgccgggatcactcacggcatggacgagctgtacaagtaagctttcccgggcatcaccatcaccatcactagctc

gaggcctttaactctggtttcattaaattttctttagtttgaatttactgttattcggtgtgcatttctatgtttggtg

agcggttttctgtgctcagagtgtgtttattttatgtaatttaatttctttgtgagctcctgtttagcaggtcgtccct

tcagcaaggacacaaaaagattttaattttattaaaaaaaaaaaaaaaaaagaccgggaattcgatatcaagcttatcg

acctgcagatcgttcaaacatttggcaataaagtttcttaagattgaatcctgttgccggtcttgcggtgattatcata

taatttctgttgaattacgttaagcatgtaataattaacatgtaatgcatgacgttatttatgagatgggtttttatga

ttagagtcccgcaattatacatttaatacgcgatagaaaacaaaatatagcgcgcaaactaggataaattatcgcgcgc

ggtgtcatctatgttactagatccgctgtcaagcgaatgattattttatgaatatatttcattgtgcaagtagatagaa

attacatatgttacataacacacgaaataaacaaaaaaagacaatccaaaaacaaacaccccaaaaaaaataatcactt

tagataaactcgtatgaggagaggcacgttaagctcagtgactcgacgattcccgagcaaaaaaagtctccccgtcaca

catatagtgggtgacgcaattatctttaaagtaatccttctgttgacttgtcattgataacatccagtcttcgtcagga

ttgcaaagaattatagaagggatcccaccttttattttcttcttttttccatatttagggttgacagtgaaatcagact

ggcaacctattaattgcttccacaatgggacgaacttgaaggggatgtcgtcgatgatattataggtggcgtgttcatc

gtagttggtgaaatcgatggtaccgttccaatagttgtgtcgtccgagacttctagcccaggtggtctttccggtacga

gttggtccgcagatgtagaggctggggtgtcggattccattccttccattgtcctggttaaatcggccatccattcaag

gtcagattgagcttgttggtatgagacaggatgtatgtaagtataagcgtctatgcttacatggtatagatgggtttcc

ctccaggagtgtagatcttcgtggcagcgaagatctgattctgtgaagggcgacacatacggttcaggttgtggaggga

ataatttgttggctgaatattccagccattgaagttttgttgcccattcatgagggaattcttccttgatcatgtcaag

atattcctccttagacgttgcagtctggataatagttctccatcgtgcgtcagatttgcgaggagataccttatgatct

cggaaatctcctctggttttaatatctccgtcctttgatatgtaatcaaggacttgtttagagtttctagctggctgga

tattagggtgatttccttcaaaatcgaaaaaagaaggatccctaatacaaggttttttatcaagctggagaagagcatg

atagtgggtagtgccatcttgatgaagctcagaagcaacaccaaggaagaaaataagaaaaggtgtgagtttctcccag

agaaactggaataaatcatctctttgagatgagcacttgggataggtaaggaaaacatatttagattggagtctgaagt

tcttactagcagaaggcattttgttgtgactccgaggggttgcctcaaactctatcttataaccggcgtggaggcatgg

aggcaggggtattttggtcattttaatagatagtggaaaatgacgtggaatttacttaaagacgaagtctttgcgacaa

gggggggcccacgccgaatttaatattaccggcgtggcccccccttatcgcgagtgctttagcacgagcggtccagatt

taaagtagaaaatttcccgcccactagggttaaaggtgttcacactatacaagcatatacgatgtgatggtattgacta

gagtttctccgct

**pGB-R-DsRed-P19**

Features:

pVS1 Sta: 1108 – 2108

pVS1-REP: 2701 – 3701

pBR322: 4111 – 4371

pBR322 ori: 4511 – 4791

Spm resistance: 5083 – 6093

Spm promoter: 6094 - 6227

35S promoter: 6711 – 7136

TMV omega: 7153 – 7214

P19: 7224 – 7742

Nos terminator: 7755 – 8007

SF: 8018 - 8052

LIR: 8065 - 8354

35S promoter: 8367 - 8681

5'UTR from CPMV RNA-2: 8682 - 9192

DsRed: 9200 - 9877

3'UTR from CPMV RNA-2: 9920 - 10103

Nos terminator: 10157 - 10409

SIR: 10422 - 10574

Rep2A: 10579 - 11013

Rep: 10794 - 11669

LIR: 11671 – 11972

cgctgtcatgagaccggatcctgacaggatatattggcgggtaaacctaagagaaaagagcgtttattagaataatcgg

atatttaaaagggcgtgaaaaggtttatccgttcgtccatttgtatgtgcatgccaaccacagggttcccctcgggatc

aaagtactttgatccaacccctccgctgctatagtgcagtcggcttctgacgttcagtgcagccgtcatctgaaaacga

catgtcgcacaagtcctaagttacgcgacaggctgccgccctgcccttttcctggcgttttcttgtcgcgtgttttagt

cgcataaagtagaatacttgcgactagaaccggagacattacgccatgaacaagagcgccgccgctggcctgctgggct

atgcccgcgtcagcaccgacgaccaggacttgaccaaccaacgggccgaactgcacgcggccggctgcaccaagctgtt

ttccgagaagatcaccggcaccaggcgcgaccgcccggagctggccaggatgcttgaccacctacgccctggcgacgtt

gtgacagtgaccaggctagaccgcctggcccgcagcacccgcgacctactggacattgccgagcgcatccaggaggccg

gcgcgggcctgcgtagcctggcagagccgtgggccgacaccaccacgccggccggccgcatggtgttgaccgtgttcgc

cggcattgccgagttcgagcgttccctaatcatcgaccgcacccggagcgggcgcgaggccgccaaggcccgaggcgtg

aagtttggcccccgccctaccctcaccccggcacagatcgcgcacgcccgcgagctgatcgaccaggaaggccgcaccg

tgaaagaggcggctgcactgcttggcgtgcatcgctcgaccctgtaccgcgcacttgagcgcagcgaggaagtgacgcc

caccgaggccaggcggcgcggtgccttccgtgaggacgcattgaccgaggccgacgccctggcggccgccgagaatgaa

cgccaagaggaacaagcatgaaaccgcaccaggacggccaggacgaaccgtttttcattaccgaagagatcgaggcgga

gatgatcgcggccgggtacgtgttcgagccgcccgcgcacctctcaaccgtgcggctgcatgaaatcctggccggtttg

tctgatgccaagctggcggcctggccggccagcttggccgctgaagaaaccgagcgccgccgtctaaaaaggtgatgtg

tatttgagtaaaacagcttgcgtcatgcggtcgctgcgtatatgatccgatgagtaaataaacaaatacgcaaggggaa

cgcatgaaggttatcgctgtacttaaccagaaaggcgggtcaggcaagacgaccatcggaacccatctagcccgcgccc

tgcaactcgccggggccgatgttctgttagtcgattccgatccccagggcagtgcccgcgattgggcggccgtgcggga

agatcaaccgctaaccgttgtcggcatcgaccgcccgacgattgaccgcgacgtgaaggccatcggccggcgcgacttc

gtagtgatcgacggagcgccccaggcggcggacttggctgtgtccgcgatcaaggcagccgacttcgtgctgattccgg

tgcagccaagcccttacgacatatgggccaccgccgacctggtggagctggttaagcagcgcattgaggtcacggatgg

aaggctacaagcggcctttgtcgtgtcgcgggcgatcaaaggcacgcgcatcggcggtgaggttgccgaggcgctggcc

gggtacgagctgcccattcttgagtcccgtatcacgcagcgcgtgagctacccaggcactgccgccgccggcacaaccg

ttcttgaatcagaacccgagggcgacgctgcccgcgaggtccaggcgctggccgctgaaattaaatcaaaactcatttg

agttaatgaggtaaagagaaaatgagcaaaagcacaaacacgctaagtgccggccgtccgagcgcacgcagcagcaagg

ctgcaacgttggccagcctggcagacacgccagccatgaagcgggtcaactttcagttgccggcggaggatcacaccaa

gctgaagatgtacgcggtacgccaaggcaagaccattaccgagctgctatctgaatagatcgcgcagctaccagagtaa

atgagcaaatgaataaatgagtagatgaattttagcggctaaaggaggcggcatggaaaatcaagaacaaccaggcacc

gacgccgtggaatgccccatgtgtggaggaacgggcggttggccaggcgtaagcggctgggttgtctgccggccctgca

atggcactggaacccccaagcccgaggaatcggcgtgacggtcgcaaaccatccggcccggtacaaatcggcgcggcgc

tgggtgatgacctggtggagaagttgaaggccgcgcaggccgcccagcggcaacgcatcgaggcagaagcacgccccgg

tgaatcgtggcaagcggccgctgatcgaatccgcaaagaatcccggcaaccgccggcagccggtgcgccgtcgattagg

aagccgcccaagggcgacgagcaaccagattttttcgttccgatgctctatgacgtgggcacccgcgatagtcgcagca

tcatggacgtggccgttttccgtctgtcgaagcgtgaccgacgagctggcgaggtgatccgctacgagcttccagacgg

gcacgtagaggtttccgcagggccggccggcatggccagtgtgtgggattacgacctggtactgatggcggtttcccat

ctaaccgaatccatgaaccgataccgggaagggaagggagacaagcccggccgcgtgttccgtccacacgttgcggacg

tactcaagttctgccggcgagccgatggcggaaagcagaaagacgacctggtagaaacctgcattcggttaaacaccac

gcacgttgccatgcagcgtacgaagaaggccaagaacggccgcctggtgacggtatccgagggtgaagccttgattagc

cgctacaagatcgtaaagagcgaaaccgggcggccggagtacatcgagatcgagctagctgattggatgtaccgcgaga

tcacagaaggcaagaacccggacgtgctgacggttcaccccgattactttttgatcgatcccggcatcggccgttttct

ctaccgcctggcacgccgcgccgcaggcaaggcagaagccagatggttgttcaagacgatctacgaacgcagtggcagc

gccggagagttcaagaagttctgtttcaccgtgcgcaagctgatcgggtcaaatgacctgccggagtacgatttgaagg

aggaggcggggcaggctggcccgatcctagtcatgcgctaccgcaacctgatcgagggcgaagcatccgccggttccta

atgtacggagcagatgctagggcaaattgccctagcaggggaaaaaggtcgaaaaggactctttcctgtggatagcacg

tacattgggaacccaaagccgtacattgggaaccggaacccgtacattgggaacccaaagccgtacattgggaaccggt

cacacatgtaagtgactgatataaaagagaaaaaaggcgatttttccgcctaaaactctttaaaacttattaaaactct

taaaacccgcctggcctgtgcataactgtctggccagcgcacagccgaagagctgcaaaaagcgcctacccttcggtcg

ctgcgctccctacgccccgccgcttcgcgtcggcctatcgcggccgctggccgctcaaaaatggctggcctacggccag

gcaatctaccagggcgcggacaagccgcgccgtcgccactcgaccgccggcgcccacatcaaggcaccctgcctcgcgc

gtttcggtgatgacggtgaaaacctctgacacatgcagctcccggtgacggtcacagcttgtctgtaagcggatgccgg

gagcagacaagcccgtcagggcgcgtcagcgggtgttggcgggtgtcggggcgcagccatgacccagtcacgtagcgat

agcggagtgtatactggcttaactatgcggcatcagagcagattgtactgagagtgcaccatatgcggtgtgaaatacc

gcacagatgcgtaaggagaaaataccgcatcaggcgctcttccgcttcctcgctcactgactcgctgcgctcggtcgtt

cggctgcggcgagcggtatcagctcactcaaaggcggtaatacggttatccacagaatcaggggataacgcaggaaaga

acatgtgagcaaaaggccagcaaaaggccaggaaccgtaaaaaggccgcgttgctggcgtttttccataggctccgccc

ccctgacgagcatcacaaaaatcgacgctcaagtcagaggtggcgaaacccgacaggactataaagataccaggcgttt

ccccctggaagctccctcgtgcgctctcctgttccgaccctgccgcttaccggatacctgtccgcctttctcccttcgg

gaagcgtggcgctttctcatagctcacgctgtaggtatctcagttcggtgtaggtcgttcgctccaagctgggctgtgt

gcacgaaccccccgttcagcccgaccgctgcgccttatccggtaactatcgtcttgagtccaacccggtaagacacgac

ttatcgccactggcagcagccactggtaacaggattagcagagcgaggtatgtaggcggtgctacagagttcttgaagt

ggtggcctaactacggctacactagaaggacagtatttggtatctgcgctctgctgaagccagttaccttcggaaaaag

agttggtagctcttgatccggcaaacaaaccaccgctggtagcggtggtttttttgtttgcaagcagcagattacgcgc

agaaaaaaaggatctcaagaagatcctttgatcttttctacggggtctgacgctcagtggaacgaaaactcacgttaag

ggattttggtcatgcattctaggtgattatttgccgactaccttggtgatctcgcctttcacgtagtggacaaattctt

ccaactgatctgcgcgcgaggccaagcgatcttcttcttgtccaagataagcctgtctagcttcaagtatgacgggctg

atactgggccggcaggcgctccattgcccagtcggcagcgacatccttcggcgcgattttgccggttactgcgctgtac

caaatgcgggacaacgtaagcactacatttcgctcatcaccagcccagtcgggcggcgagttccatagcgttaaggttt

catttagcgcctcaaatagatcctgttcaggaaccggatcaaagagttcctccgccgctggacctaccaaggcaacgct

atgttctcttgcttttgtcagcaagatagccagatcaatgtcgatcgtggctggctcgaagatacctgcaagaatgtca

ttgcgctgccattctccaaattgcagttcgcgcttagctggataacgccacggaatgatgtcgtcgtgcacaacaatgg

tgacttctacagcgcggagaatctcgctctctccaggggaagccgaagtttccaaaaggtcgttgatcaaagctcgccg

cgttgtttcatcaagccttacggtcaccgtaaccagcaaatcaatatcactgtgtggcttcaggccgccatccactgcg

gagccgtacaaatgtacggccagcaacgtcggttcgagatggcgctcgatgacgccaactacctctgatagttgagtcg

atacttcggcgatcaccgcttccctcataatgtttaactttgttttagggcgactgccctgctgcgtaacatcgttgct

gctccataacatcaaacatcgacccacggcgtaacgcgcttgctgcttggatgcccgaggcatagactgtaccccaaaa

aaacagtcataacaagccatgaaaaccgccactgcgccgttaccaccgctgcgttcggtcaaggttctggaccagttgc

gtgagcgcatacgctacttgcattacagcttacgaaccgaacaggcttatgtccactgggttcgtgccttcatccgttt

ccacggtgtgcgtcacccggcaaccttgggtagcagcgaagtcgaggcatttctgtcctggctggaacagaacttatta

tttccttcctcttttctacagtatttaaagataccccaagaagctaattataacaagacgaactccaattcactgttcc

ttgcattctaaaaccttaaataccagaaaacagctttttcaaagttgttttcaaagttggcgtataacatagtatcgac

ggagccgattttgaaaccgcggtgatcacaggcagcaacgctctgtcatcgttacaatcaacatgctaccctccgcgag

atcatccgtgtttcaaacccggcagcttagttgccgttcttccgaatagcatcggtaacatgagcaaagtctgccgcct

tacaacggctctcccgctgacgccgtcccggactgatgggctgcctgtatcgagtggtgattttgtgccgagctgccgg

tcggggagctgttggctggctggtggcaggatatattgtggtgtaaacataacggatccggtctcaggagggaggtcaa

catggtggagcacgacactctggtctactccaaaaatgtcaaagatacagtctcagaagatcaaagggctattgagact

tttcaacaaaggataatttcgggaaacctcctcggattccattgcccagctatctgtcacttcatcgaaaggacagtag

aaaaggaaggtggctcctacaaatgccatcattgcgataaaggaaaggctatcattcaagatctctctgccgacagtgg

tcccaaagatggacccccacccacgaggagcatcgtggaaaaagaagaggttccaaccacgtctacaaagcaagtggat

tgatgtgacatctccactgacgtaagggatgacgcacaatcccactatccttcgcaagacccttcctctatataaggaa

gttcatttcatttggagaggacacgctcgagtataagagctcatttttacaacaattaccaacaacaacaaacaacaaa

caacattacaattacatttacaattatcgatacaatggaacgagctatacaaggaaacgacgctagggaacaagctaac

agtgaacgttgggatggaggatcaggaggtaccacttctcccttcaaacttcctgacgaaagtccgagttggactgagt

ggcggctacataacgatgagactaattcgaatcaagataatccccttggtttcaaggaaagctggggtttcgggaaagt

tgtatttaagagatatctcagatacgacaggacggaagcttcactgcacagagtccttggatcttggacgggagattcg

gttaactatgcagcatctcgatttttcggtttcgaccagatcggatgtacctatagtattcggtttcgaggagttagta

tcaccgtttctggaggctctcgaactcttcagcatctctgtgagatggcaattcggtctaagcaagaactgctacagct

tgccccaatcgaagtggaaagtaatgtatcaagaggatgccctgaaggtactgaaaccttcgaaaaagaaagcgagtga

gcttgtcaagcagatcgttcaaacatttggcaataaagtttcttaagattgaatcctgttgccggtcttgcgatgatta

tcatataatttctgttgaattacgttaagcatgtaataattaacatgtaatgcatgacgttatttatgagatgggtttt

tatgattagagtcccgcaattatacatttaatacgcgatagaaaacaaaatatagcgcgcaaactaggataaattatcg

cgcgcggtgtcatctatgttactagatcgacgctccatggagccctcaattgtactaccatcatttcttgttccgctgc

ttggaggtgactccgaggggttgcctcaaactctatcttataaccggcgtggaggcatggaggcaggggtattttggtc

attttaatagatagtggaaaatgacgtggaatttacttaaagacgaagtctttgcgacaagggggggcccacgccgaat

ttaatattaccggcgtggcccccccttatcgcgagtgctttagcacgagcggtccagatttaaagtagaaaatttcccg

cccactagggttaaaggtgttcacactataaaagcatatacgatgtgatggtatttgctcgctggtaggagggaaacct

cctcggattccattgcccagctatctgtcactttattgagaagatagtggaaaaggaaggtggctcctacaaatgccat

cattgcgataaaggaaaggccatcgttgaagatgcctctgccgacagtggtcccaaagatggacccccacccacgagga

gcatcgtggaaaaagaagacgttccaaccacgtcttcaaagcaagtggattgatgtgatatctccactgacgtaaggga

tgacgcacaatcccactatccttcgcaagacccttcctctatataaggaagttcatttcatttggagaggtattaaaat

cttaataggttttgataaaagcgaacgtggggaaacccgaaccaaaccttcttctaaactctctctcatctctcttaaa

gcaaacttctctcttgtctttcttgcgtgagcgatcttcaacgttgtcagatcgtgcttcggcaccagtacaacgtttt

ctttcactgaagcgaaatcaaagatctctttgtggacacgtagtgcggcgccattaaataacgtgtacttgtcctattc

ttgtcggtgtggtcttgggaaaagaaagcttgctggaggctgctgttcagccccatacattacttgttacgattctgct

gactttcggcgggtgcaatatctctacttctgcttgacgaggtattgttgcctgtacttctttcttcttcttcttgctg

attggttctataagaaatctagtattttctttgaaacagagttttcccgtggttttcgaacttggagaaagattgttaa

gcttctgtatattctgcccaaattcgcgaccggtaatggggtcatccaagaatgttatcaaggagttcatgaggtttaa

ggttcgcatggaaggaacggtcaatgggcacgagtttgaaatagaaggcgaaggagaggggaggccatacgaaggccac

aataccgtaaagcttaaggtaaccaaggggggacctttgccatttgcttgggatattttgtcaccacaatttcagtatg

gaagcaaggtatatgtcaagcaccctgccgacataccagactataaaaagctgtcatttcctgaaggatttaaatggga

aagggtcatgaactttgaagatggtggcgtcgttactgtaacccaggattccagtttgcaggatggctgtttcatctac

aaggtcaagttcattggcgtgaactttccttccgatggacctgttatgcaaaagaaaacaatgggctgggaagccagca

ctgagcgtttgtatcctcgtgatggcgtgttgaaaggagagattcataaggctctgaagctgaaagacggtggtcatta

cctagttgaattcaaaagtatttacatggcaaagaagcctgtgcagctaccagggtactactatgttgactccaaactg

gatataacaagccacaacgaagattatacaatcgttgagcagtatgaaagaaccgagggacgccaccatctgttccttt

aagctttcccgggcatcaccatcaccatcactagctcgaggcctttaactctggtttcattaaattttctttagtttga

atttactgttattcggtgtgcatttctatgtttggtgagcggttttctgtgctcagagtgtgtttattttatgtaattt

aatttctttgtgagctcctgtttagcaggtcgtcccttcagcaaggacacaaaaagattttaattttattaaaaaaaaa

aaaaaaaaagaccgggaattcgatatcaagcttatcgacctgcagatcgttcaaacatttggcaataaagtttcttaag

attgaatcctgttgccggtcttgcggtgattatcatataatttctgttgaattacgttaagcatgtaataattaacatg

taatgcatgacgttatttatgagatgggtttttatgattagagtcccgcaattatacatttaatacgcgatagaaaaca

aaatatagcgcgcaaactaggataaattatcgcgcgcggtgtcatctatgttactagatccgctgtcaagcgaatgatt

attttatgaatatatttcattgtgcaagtagatagaaattacatatgttacataacacacgaaataaacaaaaaaagac

aatccaaaaacaaacaccccaaaaaaaataatcactttagataaactcgtatgaggagaggcacgttaagctcagtgac

tcgacgattcccgagcaaaaaaagtctccccgtcacacatatagtgggtgacgcaattatctttaaagtaatccttctg

ttgacttgtcattgataacatccagtcttcgtcaggattgcaaagaattatagaagggatcccaccttttattttcttc

ttttttccatatttagggttgacagtgaaatcagactggcaacctattaattgcttccacaatgggacgaacttgaagg

ggatgtcgtcgatgatattataggtggcgtgttcatcgtagttggtgaaatcgatggtaccgttccaatagttgtgtcg

tccgagacttctagcccaggtggtctttccggtacgagttggtccgcagatgtagaggctggggtgtcggattccattc

cttccattgtcctggttaaatcggccatccattcaaggtcagattgagcttgttggtatgagacaggatgtatgtaagt

ataagcgtctatgcttacatggtatagatgggtttccctccaggagtgtagatcttcgtggcagcgaagatctgattct

gtgaagggcgacacatacggttcaggttgtggagggaataatttgttggctgaatattccagccattgaagttttgttg

cccattcatgagggaattcttccttgatcatgtcaagatattcctccttagacgttgcagtctggataatagttctcca

tcgtgcgtcagatttgcgaggagataccttatgatctcggaaatctcctctggttttaatatctccgtcctttgatatg

taatcaaggacttgtttagagtttctagctggctggatattagggtgatttccttcaaaatcgaaaaaagaaggatccc

taatacaaggttttttatcaagctggagaagagcatgatagtgggtagtgccatcttgatgaagctcagaagcaacacc

aaggaagaaaataagaaaaggtgtgagtttctcccagagaaactggaataaatcatctctttgagatgagcacttggga

taggtaaggaaaacatatttagattggagtctgaagttcttactagcagaaggcattttgttgtgactccgaggggttg

cctcaaactctatcttataaccggcgtggaggcatggaggcaggggtattttggtcattttaatagatagtggaaaatg

acgtggaatttacttaaagacgaagtctttgcgacaagggggggcccacgccgaatttaatattaccggcgtggccccc

ccttatcgcgagtgctttagcacgagcggtccagatttaaagtagaaaatttcccgcccactagggttaaaggtgttca

cactatacaagcatatacgatgtgatggtattgactagagtttctccgct

**pGB-R-GFP-DsRed**

Features:

pVS1 Sta: 1108 – 2108

pVS1-REP: 2701 – 3701

pBR322: 4111 – 4371

pBR322 ori: 4511 – 4791

Spm resistance: 5083 – 6093

Spm promoter: 6094 - 6227

35S promoter: 6711 – 7136

TMV omega: 7153 – 7214

P19: 7224 – 7742

Nos terminator: 7755 – 8007

LIR: 8022 - 8311

35S promoter: 8324 - 8638

5'UTR from CPMV RNA-2: 8639 - 9149

GFP: 9157 - 9876

3'UTR from CPMV RNA-2: 9919 - 10102

Nos terminator: 10156 - 10408

35S promoter: 10421 - 10735

5'UTR from CPMV RNA-2: 10736 - 11246

DsRed: 11254 - 11931

3'UTR from CPMV RNA-2: 11974 - 12157

Nos terminator: 12211 - 12463

SIR: 12476 - 12628

Rep2A: 12633 – 13067

Rep: 12848 - 13723

LIR: 13725 - 14026

cgctgtcatgagaccggatcctgacaggatatattggcgggtaaacctaagagaaaagagcgtttattagaataatcgg

atatttaaaagggcgtgaaaaggtttatccgttcgtccatttgtatgtgcatgccaaccacagggttcccctcgggatc

aaagtactttgatccaacccctccgctgctatagtgcagtcggcttctgacgttcagtgcagccgtcatctgaaaacga

catgtcgcacaagtcctaagttacgcgacaggctgccgccctgcccttttcctggcgttttcttgtcgcgtgttttagt

cgcataaagtagaatacttgcgactagaaccggagacattacgccatgaacaagagcgccgccgctggcctgctgggct

atgcccgcgtcagcaccgacgaccaggacttgaccaaccaacgggccgaactgcacgcggccggctgcaccaagctgtt

ttccgagaagatcaccggcaccaggcgcgaccgcccggagctggccaggatgcttgaccacctacgccctggcgacgtt

gtgacagtgaccaggctagaccgcctggcccgcagcacccgcgacctactggacattgccgagcgcatccaggaggccg

gcgcgggcctgcgtagcctggcagagccgtgggccgacaccaccacgccggccggccgcatggtgttgaccgtgttcgc

cggcattgccgagttcgagcgttccctaatcatcgaccgcacccggagcgggcgcgaggccgccaaggcccgaggcgtg

aagtttggcccccgccctaccctcaccccggcacagatcgcgcacgcccgcgagctgatcgaccaggaaggccgcaccg

tgaaagaggcggctgcactgcttggcgtgcatcgctcgaccctgtaccgcgcacttgagcgcagcgaggaagtgacgcc

caccgaggccaggcggcgcggtgccttccgtgaggacgcattgaccgaggccgacgccctggcggccgccgagaatgaa

cgccaagaggaacaagcatgaaaccgcaccaggacggccaggacgaaccgtttttcattaccgaagagatcgaggcgga

gatgatcgcggccgggtacgtgttcgagccgcccgcgcacctctcaaccgtgcggctgcatgaaatcctggccggtttg

tctgatgccaagctggcggcctggccggccagcttggccgctgaagaaaccgagcgccgccgtctaaaaaggtgatgtg

tatttgagtaaaacagcttgcgtcatgcggtcgctgcgtatatgatccgatgagtaaataaacaaatacgcaaggggaa

cgcatgaaggttatcgctgtacttaaccagaaaggcgggtcaggcaagacgaccatcggaacccatctagcccgcgccc

tgcaactcgccggggccgatgttctgttagtcgattccgatccccagggcagtgcccgcgattgggcggccgtgcggga

agatcaaccgctaaccgttgtcggcatcgaccgcccgacgattgaccgcgacgtgaaggccatcggccggcgcgacttc

gtagtgatcgacggagcgccccaggcggcggacttggctgtgtccgcgatcaaggcagccgacttcgtgctgattccgg

tgcagccaagcccttacgacatatgggccaccgccgacctggtggagctggttaagcagcgcattgaggtcacggatgg

aaggctacaagcggcctttgtcgtgtcgcgggcgatcaaaggcacgcgcatcggcggtgaggttgccgaggcgctggcc

gggtacgagctgcccattcttgagtcccgtatcacgcagcgcgtgagctacccaggcactgccgccgccggcacaaccg

ttcttgaatcagaacccgagggcgacgctgcccgcgaggtccaggcgctggccgctgaaattaaatcaaaactcatttg

agttaatgaggtaaagagaaaatgagcaaaagcacaaacacgctaagtgccggccgtccgagcgcacgcagcagcaagg

ctgcaacgttggccagcctggcagacacgccagccatgaagcgggtcaactttcagttgccggcggaggatcacaccaa

gctgaagatgtacgcggtacgccaaggcaagaccattaccgagctgctatctgaatagatcgcgcagctaccagagtaa

atgagcaaatgaataaatgagtagatgaattttagcggctaaaggaggcggcatggaaaatcaagaacaaccaggcacc

gacgccgtggaatgccccatgtgtggaggaacgggcggttggccaggcgtaagcggctgggttgtctgccggccctgca

atggcactggaacccccaagcccgaggaatcggcgtgacggtcgcaaaccatccggcccggtacaaatcggcgcggcgc

tgggtgatgacctggtggagaagttgaaggccgcgcaggccgcccagcggcaacgcatcgaggcagaagcacgccccgg

tgaatcgtggcaagcggccgctgatcgaatccgcaaagaatcccggcaaccgccggcagccggtgcgccgtcgattagg

aagccgcccaagggcgacgagcaaccagattttttcgttccgatgctctatgacgtgggcacccgcgatagtcgcagca

tcatggacgtggccgttttccgtctgtcgaagcgtgaccgacgagctggcgaggtgatccgctacgagcttccagacgg

gcacgtagaggtttccgcagggccggccggcatggccagtgtgtgggattacgacctggtactgatggcggtttcccat

ctaaccgaatccatgaaccgataccgggaagggaagggagacaagcccggccgcgtgttccgtccacacgttgcggacg

tactcaagttctgccggcgagccgatggcggaaagcagaaagacgacctggtagaaacctgcattcggttaaacaccac

gcacgttgccatgcagcgtacgaagaaggccaagaacggccgcctggtgacggtatccgagggtgaagccttgattagc

cgctacaagatcgtaaagagcgaaaccgggcggccggagtacatcgagatcgagctagctgattggatgtaccgcgaga

tcacagaaggcaagaacccggacgtgctgacggttcaccccgattactttttgatcgatcccggcatcggccgttttct

ctaccgcctggcacgccgcgccgcaggcaaggcagaagccagatggttgttcaagacgatctacgaacgcagtggcagc

gccggagagttcaagaagttctgtttcaccgtgcgcaagctgatcgggtcaaatgacctgccggagtacgatttgaagg

aggaggcggggcaggctggcccgatcctagtcatgcgctaccgcaacctgatcgagggcgaagcatccgccggttccta

atgtacggagcagatgctagggcaaattgccctagcaggggaaaaaggtcgaaaaggactctttcctgtggatagcacg

tacattgggaacccaaagccgtacattgggaaccggaacccgtacattgggaacccaaagccgtacattgggaaccggt

cacacatgtaagtgactgatataaaagagaaaaaaggcgatttttccgcctaaaactctttaaaacttattaaaactct

taaaacccgcctggcctgtgcataactgtctggccagcgcacagccgaagagctgcaaaaagcgcctacccttcggtcg

ctgcgctccctacgccccgccgcttcgcgtcggcctatcgcggccgctggccgctcaaaaatggctggcctacggccag

gcaatctaccagggcgcggacaagccgcgccgtcgccactcgaccgccggcgcccacatcaaggcaccctgcctcgcgc

gtttcggtgatgacggtgaaaacctctgacacatgcagctcccggtgacggtcacagcttgtctgtaagcggatgccgg

gagcagacaagcccgtcagggcgcgtcagcgggtgttggcgggtgtcggggcgcagccatgacccagtcacgtagcgat

agcggagtgtatactggcttaactatgcggcatcagagcagattgtactgagagtgcaccatatgcggtgtgaaatacc

gcacagatgcgtaaggagaaaataccgcatcaggcgctcttccgcttcctcgctcactgactcgctgcgctcggtcgtt

cggctgcggcgagcggtatcagctcactcaaaggcggtaatacggttatccacagaatcaggggataacgcaggaaaga

acatgtgagcaaaaggccagcaaaaggccaggaaccgtaaaaaggccgcgttgctggcgtttttccataggctccgccc

ccctgacgagcatcacaaaaatcgacgctcaagtcagaggtggcgaaacccgacaggactataaagataccaggcgttt

ccccctggaagctccctcgtgcgctctcctgttccgaccctgccgcttaccggatacctgtccgcctttctcccttcgg

gaagcgtggcgctttctcatagctcacgctgtaggtatctcagttcggtgtaggtcgttcgctccaagctgggctgtgt

gcacgaaccccccgttcagcccgaccgctgcgccttatccggtaactatcgtcttgagtccaacccggtaagacacgac

ttatcgccactggcagcagccactggtaacaggattagcagagcgaggtatgtaggcggtgctacagagttcttgaagt

ggtggcctaactacggctacactagaaggacagtatttggtatctgcgctctgctgaagccagttaccttcggaaaaag

agttggtagctcttgatccggcaaacaaaccaccgctggtagcggtggtttttttgtttgcaagcagcagattacgcgc

agaaaaaaaggatctcaagaagatcctttgatcttttctacggggtctgacgctcagtggaacgaaaactcacgttaag

ggattttggtcatgcattctaggtgattatttgccgactaccttggtgatctcgcctttcacgtagtggacaaattctt

ccaactgatctgcgcgcgaggccaagcgatcttcttcttgtccaagataagcctgtctagcttcaagtatgacgggctg

atactgggccggcaggcgctccattgcccagtcggcagcgacatccttcggcgcgattttgccggttactgcgctgtac

caaatgcgggacaacgtaagcactacatttcgctcatcaccagcccagtcgggcggcgagttccatagcgttaaggttt

catttagcgcctcaaatagatcctgttcaggaaccggatcaaagagttcctccgccgctggacctaccaaggcaacgct

atgttctcttgcttttgtcagcaagatagccagatcaatgtcgatcgtggctggctcgaagatacctgcaagaatgtca

ttgcgctgccattctccaaattgcagttcgcgcttagctggataacgccacggaatgatgtcgtcgtgcacaacaatgg

tgacttctacagcgcggagaatctcgctctctccaggggaagccgaagtttccaaaaggtcgttgatcaaagctcgccg

cgttgtttcatcaagccttacggtcaccgtaaccagcaaatcaatatcactgtgtggcttcaggccgccatccactgcg

gagccgtacaaatgtacggccagcaacgtcggttcgagatggcgctcgatgacgccaactacctctgatagttgagtcg

atacttcggcgatcaccgcttccctcataatgtttaactttgttttagggcgactgccctgctgcgtaacatcgttgct

gctccataacatcaaacatcgacccacggcgtaacgcgcttgctgcttggatgcccgaggcatagactgtaccccaaaa

aaacagtcataacaagccatgaaaaccgccactgcgccgttaccaccgctgcgttcggtcaaggttctggaccagttgc

gtgagcgcatacgctacttgcattacagcttacgaaccgaacaggcttatgtccactgggttcgtgccttcatccgttt

ccacggtgtgcgtcacccggcaaccttgggtagcagcgaagtcgaggcatttctgtcctggctggaacagaacttatta

tttccttcctcttttctacagtatttaaagataccccaagaagctaattataacaagacgaactccaattcactgttcc

ttgcattctaaaaccttaaataccagaaaacagctttttcaaagttgttttcaaagttggcgtataacatagtatcgac

ggagccgattttgaaaccgcggtgatcacaggcagcaacgctctgtcatcgttacaatcaacatgctaccctccgcgag

atcatccgtgtttcaaacccggcagcttagttgccgttcttccgaatagcatcggtaacatgagcaaagtctgccgcct

tacaacggctctcccgctgacgccgtcccggactgatgggctgcctgtatcgagtggtgattttgtgccgagctgccgg

tcggggagctgttggctggctggtggcaggatatattgtggtgtaaacataacggatccggtctcaggagggaggtcaa

catggtggagcacgacactctggtctactccaaaaatgtcaaagatacagtctcagaagatcaaagggctattgagact

tttcaacaaaggataatttcgggaaacctcctcggattccattgcccagctatctgtcacttcatcgaaaggacagtag

aaaaggaaggtggctcctacaaatgccatcattgcgataaaggaaaggctatcattcaagatctctctgccgacagtgg

tcccaaagatggacccccacccacgaggagcatcgtggaaaaagaagaggttccaaccacgtctacaaagcaagtggat

tgatgtgacatctccactgacgtaagggatgacgcacaatcccactatccttcgcaagacccttcctctatataaggaa

gttcatttcatttggagaggacacgctcgagtataagagctcatttttacaacaattaccaacaacaacaaacaacaaa

caacattacaattacatttacaattatcgatacaatggaacgagctatacaaggaaacgacgctagggaacaagctaac

agtgaacgttgggatggaggatcaggaggtaccacttctcccttcaaacttcctgacgaaagtccgagttggactgagt

ggcggctacataacgatgagactaattcgaatcaagataatccccttggtttcaaggaaagctggggtttcgggaaagt

tgtatttaagagatatctcagatacgacaggacggaagcttcactgcacagagtccttggatcttggacgggagattcg

gttaactatgcagcatctcgatttttcggtttcgaccagatcggatgtacctatagtattcggtttcgaggagttagta

tcaccgtttctggaggctctcgaactcttcagcatctctgtgagatggcaattcggtctaagcaagaactgctacagct

tgccccaatcgaagtggaaagtaatgtatcaagaggatgccctgaaggtactgaaaccttcgaaaaagaaagcgagtga

gcttgtcaagcagatcgttcaaacatttggcaataaagtttcttaagattgaatcctgttgccggtcttgcgatgatta

tcatataatttctgttgaattacgttaagcatgtaataattaacatgtaatgcatgacgttatttatgagatgggtttt

tatgattagagtcccgcaattatacatttaatacgcgatagaaaacaaaatatagcgcgcaaactaggataaattatcg

cgcgcggtgtcatctatgttactagatcgacgctccatggaggtgactccgaggggttgcctcaaactctatcttataa

ccggcgtggaggcatggaggcaggggtattttggtcattttaatagatagtggaaaatgacgtggaatttacttaaaga

cgaagtctttgcgacaagggggggcccacgccgaatttaatattaccggcgtggcccccccttatcgcgagtgctttag

cacgagcggtccagatttaaagtagaaaatttcccgcccactagggttaaaggtgttcacactataaaagcatatacga

tgtgatggtatttgctcgctgcttggagggaaacctcctcggattccattgcccagctatctgtcactttattgagaag

atagtggaaaaggaaggtggctcctacaaatgccatcattgcgataaaggaaaggccatcgttgaagatgcctctgccg

acagtggtcccaaagatggacccccacccacgaggagcatcgtggaaaaagaagacgttccaaccacgtcttcaaagca

agtggattgatgtgatatctccactgacgtaagggatgacgcacaatcccactatccttcgcaagacccttcctctata

taaggaagttcatttcatttggagaggtattaaaatcttaataggttttgataaaagcgaacgtggggaaacccgaacc

aaaccttcttctaaactctctctcatctctcttaaagcaaacttctctcttgtctttcttgcgtgagcgatcttcaacg

ttgtcagatcgtgcttcggcaccagtacaacgttttctttcactgaagcgaaatcaaagatctctttgtggacacgtag

tgcggcgccattaaataacgtgtacttgtcctattcttgtcggtgtggtcttgggaaaagaaagcttgctggaggctgc

tgttcagccccatacattacttgttacgattctgctgactttcggcgggtgcaatatctctacttctgcttgacgaggt

attgttgcctgtacttctttcttcttcttcttgctgattggttctataagaaatctagtattttctttgaaacagagtt

ttcccgtggttttcgaacttggagaaagattgttaagcttctgtatattctgcccaaattcgcgaccggtaatggtgag

caagggcgaggagctgttcaccggggtggtgcccatcctggtcgagctggacggcgacgtaaacggccacaagttcagc

gtgtccggcgagggcgagggcgatgccacctacggcaagctgaccctgaagttcatctgcaccaccggcaagctgcccg

tgccctggcccaccctcgtgaccaccttcagctacggcgtgcagtgcttcagccgctaccccgaccacatgaagcagca

cgacttcttcaagtccgccatgcccgaaggctacgtccaggagcgcaccatcttcttcaaggacgacggcaactacaag

acccgcgccgaggtgaagttcgagggcgacaccctggtgaaccgcatcgagctgaagggcatcgacttcaaggaggacg

gcaacatcctggggcacaagctggagtacaactacaacagccacaacgtctatatcatggccgacaagcagaagaacgg

catcaaggtgaacttcaagatccgccacaacatcgaggacggcagcgtgcagctcgccgaccactaccagcagaacacc

cccatcggcgacggccccgtgctgctgcccgacaaccactacctgagcacccagtccgccctgagcaaagaccccaacg

agaagcgcgatcacatggtcctgctggagttcgtgaccgccgccgggatcactcacggcatggacgagctgtacaagta

agctttcccgggcatcaccatcaccatcactagctcgaggcctttaactctggtttcattaaattttctttagtttgaa

tttactgttattcggtgtgcatttctatgtttggtgagcggttttctgtgctcagagtgtgtttattttatgtaattta

atttctttgtgagctcctgtttagcaggtcgtcccttcagcaaggacacaaaaagattttaattttattaaaaaaaaaa

aaaaaaaagaccgggaattcgatatcaagcttatcgacctgcagatcgttcaaacatttggcaataaagtttcttaaga

ttgaatcctgttgccggtcttgcggtgattatcatataatttctgttgaattacgttaagcatgtaataattaacatgt

aatgcatgacgttatttatgagatgggtttttatgattagagtcccgcaattatacatttaatacgcgatagaaaacaa

aatatagcgcgcaaactaggataaattatcgcgcgcggtgtcatctatgttactagatccgctggtaggagggaaacct

cctcggattccattgcccagctatctgtcactttattgagaagatagtggaaaaggaaggtggctcctacaaatgccat

cattgcgataaaggaaaggccatcgttgaagatgcctctgccgacagtggtcccaaagatggacccccacccacgagga

gcatcgtggaaaaagaagacgttccaaccacgtcttcaaagcaagtggattgatgtgatatctccactgacgtaaggga

tgacgcacaatcccactatccttcgcaagacccttcctctatataaggaagttcatttcatttggagaggtattaaaat

cttaataggttttgataaaagcgaacgtggggaaacccgaaccaaaccttcttctaaactctctctcatctctcttaaa

gcaaacttctctcttgtctttcttgcgtgagcgatcttcaacgttgtcagatcgtgcttcggcaccagtacaacgtttt

ctttcactgaagcgaaatcaaagatctctttgtggacacgtagtgcggcgccattaaataacgtgtacttgtcctattc

ttgtcggtgtggtcttgggaaaagaaagcttgctggaggctgctgttcagccccatacattacttgttacgattctgct

gactttcggcgggtgcaatatctctacttctgcttgacgaggtattgttgcctgtacttctttcttcttcttcttgctg

attggttctataagaaatctagtattttctttgaaacagagttttcccgtggttttcgaacttggagaaagattgttaa

gcttctgtatattctgcccaaattcgcgaccggtaatggggtcatccaagaatgttatcaaggagttcatgaggtttaa

ggttcgcatggaaggaacggtcaatgggcacgagtttgaaatagaaggcgaaggagaggggaggccatacgaaggccac

aataccgtaaagcttaaggtaaccaaggggggacctttgccatttgcttgggatattttgtcaccacaatttcagtatg

gaagcaaggtatatgtcaagcaccctgccgacataccagactataaaaagctgtcatttcctgaaggatttaaatggga

aagggtcatgaactttgaagatggtggcgtcgttactgtaacccaggattccagtttgcaggatggctgtttcatctac

aaggtcaagttcattggcgtgaactttccttccgatggacctgttatgcaaaagaaaacaatgggctgggaagccagca

ctgagcgtttgtatcctcgtgatggcgtgttgaaaggagagattcataaggctctgaagctgaaagacggtggtcatta

cctagttgaattcaaaagtatttacatggcaaagaagcctgtgcagctaccagggtactactatgttgactccaaactg

gatataacaagccacaacgaagattatacaatcgttgagcagtatgaaagaaccgagggacgccaccatctgttccttt

aagctttcccgggcatcaccatcaccatcactagctcgaggcctttaactctggtttcattaaattttctttagtttga

atttactgttattcggtgtgcatttctatgtttggtgagcggttttctgtgctcagagtgtgtttattttatgtaattt

aatttctttgtgagctcctgtttagcaggtcgtcccttcagcaaggacacaaaaagattttaattttattaaaaaaaaa

aaaaaaaaagaccgggaattcgatatcaagcttatcgacctgcagatcgttcaaacatttggcaataaagtttcttaag

attgaatcctgttgccggtcttgcggtgattatcatataatttctgttgaattacgttaagcatgtaataattaacatg

taatgcatgacgttatttatgagatgggtttttatgattagagtcccgcaattatacatttaatacgcgatagaaaaca

aaatatagcgcgcaaactaggataaattatcgcgcgcggtgtcatctatgttactagatccgctgtcaagcgaatgatt

attttatgaatatatttcattgtgcaagtagatagaaattacatatgttacataacacacgaaataaacaaaaaaagac

aatccaaaaacaaacaccccaaaaaaaataatcactttagataaactcgtatgaggagaggcacgttaagctcagtgac

tcgacgattcccgagcaaaaaaagtctccccgtcacacatatagtgggtgacgcaattatctttaaagtaatccttctg

ttgacttgtcattgataacatccagtcttcgtcaggattgcaaagaattatagaagggatcccaccttttattttcttc

ttttttccatatttagggttgacagtgaaatcagactggcaacctattaattgcttccacaatgggacgaacttgaagg

ggatgtcgtcgatgatattataggtggcgtgttcatcgtagttggtgaaatcgatggtaccgttccaatagttgtgtcg

tccgagacttctagcccaggtggtctttccggtacgagttggtccgcagatgtagaggctggggtgtcggattccattc

cttccattgtcctggttaaatcggccatccattcaaggtcagattgagcttgttggtatgagacaggatgtatgtaagt

ataagcgtctatgcttacatggtatagatgggtttccctccaggagtgtagatcttcgtggcagcgaagatctgattct

gtgaagggcgacacatacggttcaggttgtggagggaataatttgttggctgaatattccagccattgaagttttgttg

cccattcatgagggaattcttccttgatcatgtcaagatattcctccttagacgttgcagtctggataatagttctcca

tcgtgcgtcagatttgcgaggagataccttatgatctcggaaatctcctctggttttaatatctccgtcctttgatatg

taatcaaggacttgtttagagtttctagctggctggatattagggtgatttccttcaaaatcgaaaaaagaaggatccc

taatacaaggttttttatcaagctggagaagagcatgatagtgggtagtgccatcttgatgaagctcagaagcaacacc

aaggaagaaaataagaaaaggtgtgagtttctcccagagaaactggaataaatcatctctttgagatgagcacttggga

taggtaaggaaaacatatttagattggagtctgaagttcttactagcagaaggcattttgttgtgactccgaggggttg

cctcaaactctatcttataaccggcgtggaggcatggaggcaggggtattttggtcattttaatagatagtggaaaatg

acgtggaatttacttaaagacgaagtctttgcgacaagggggggcccacgccgaatttaatattaccggcgtggccccc

ccttatcgcgagtgctttagcacgagcggtccagatttaaagtagaaaatttcccgcccactagggttaaaggtgttca

cactatacaagcatatacgatgtgatggtattgactagagtttctccgct

**pGB-R-DsRed-GFP**

Features:

pVS1 Sta: 1108 – 2108

pVS1-REP: 2701 – 3701

pBR322: 4111 – 4371

pBR322 ori: 4511 – 4791

Spm resistance: 5083 – 6093

Spm promoter: 6094 - 6227

35S promoter: 6711 – 7136

TMV omega: 7153 – 7214

P19: 7224 – 7742

Nos terminator: 7755 – 8007

LIR: 8022 - 8311

35S promoter: 8324 - 8638

5'UTR from CPMV RNA-2: 8639 - 9149

DsRed: 9157 - 9834

3'UTR from CPMV RNA-2: 9877 - 10060

Nos terminator: 10114 - 10366

35S promoter: 10379 - 10693

5'UTR from CPMV RNA-2: 10694 - 11204

GFP: 11212 - 11931

3'UTR from CPMV RNA-2: 11974 - 12157

Nos terminator: 12211 - 12463

SIR: 12476 - 12628

Rep2A: 12633 – 13067

Rep: 12848 - 13723

LIR: 13725 - 14026

cgctgtcatgagaccggatcctgacaggatatattggcgggtaaacctaagagaaaagagcgtttattagaataatcgg

atatttaaaagggcgtgaaaaggtttatccgttcgtccatttgtatgtgcatgccaaccacagggttcccctcgggatc

aaagtactttgatccaacccctccgctgctatagtgcagtcggcttctgacgttcagtgcagccgtcatctgaaaacga

catgtcgcacaagtcctaagttacgcgacaggctgccgccctgcccttttcctggcgttttcttgtcgcgtgttttagt

cgcataaagtagaatacttgcgactagaaccggagacattacgccatgaacaagagcgccgccgctggcctgctgggct

atgcccgcgtcagcaccgacgaccaggacttgaccaaccaacgggccgaactgcacgcggccggctgcaccaagctgtt

ttccgagaagatcaccggcaccaggcgcgaccgcccggagctggccaggatgcttgaccacctacgccctggcgacgtt

gtgacagtgaccaggctagaccgcctggcccgcagcacccgcgacctactggacattgccgagcgcatccaggaggccg

gcgcgggcctgcgtagcctggcagagccgtgggccgacaccaccacgccggccggccgcatggtgttgaccgtgttcgc

cggcattgccgagttcgagcgttccctaatcatcgaccgcacccggagcgggcgcgaggccgccaaggcccgaggcgtg

aagtttggcccccgccctaccctcaccccggcacagatcgcgcacgcccgcgagctgatcgaccaggaaggccgcaccg

tgaaagaggcggctgcactgcttggcgtgcatcgctcgaccctgtaccgcgcacttgagcgcagcgaggaagtgacgcc

caccgaggccaggcggcgcggtgccttccgtgaggacgcattgaccgaggccgacgccctggcggccgccgagaatgaa

cgccaagaggaacaagcatgaaaccgcaccaggacggccaggacgaaccgtttttcattaccgaagagatcgaggcgga

gatgatcgcggccgggtacgtgttcgagccgcccgcgcacctctcaaccgtgcggctgcatgaaatcctggccggtttg

tctgatgccaagctggcggcctggccggccagcttggccgctgaagaaaccgagcgccgccgtctaaaaaggtgatgtg

tatttgagtaaaacagcttgcgtcatgcggtcgctgcgtatatgatccgatgagtaaataaacaaatacgcaaggggaa

cgcatgaaggttatcgctgtacttaaccagaaaggcgggtcaggcaagacgaccatcggaacccatctagcccgcgccc

tgcaactcgccggggccgatgttctgttagtcgattccgatccccagggcagtgcccgcgattgggcggccgtgcggga

agatcaaccgctaaccgttgtcggcatcgaccgcccgacgattgaccgcgacgtgaaggccatcggccggcgcgacttc

gtagtgatcgacggagcgccccaggcggcggacttggctgtgtccgcgatcaaggcagccgacttcgtgctgattccgg

tgcagccaagcccttacgacatatgggccaccgccgacctggtggagctggttaagcagcgcattgaggtcacggatgg

aaggctacaagcggcctttgtcgtgtcgcgggcgatcaaaggcacgcgcatcggcggtgaggttgccgaggcgctggcc

gggtacgagctgcccattcttgagtcccgtatcacgcagcgcgtgagctacccaggcactgccgccgccggcacaaccg

ttcttgaatcagaacccgagggcgacgctgcccgcgaggtccaggcgctggccgctgaaattaaatcaaaactcatttg

agttaatgaggtaaagagaaaatgagcaaaagcacaaacacgctaagtgccggccgtccgagcgcacgcagcagcaagg

ctgcaacgttggccagcctggcagacacgccagccatgaagcgggtcaactttcagttgccggcggaggatcacaccaa

gctgaagatgtacgcggtacgccaaggcaagaccattaccgagctgctatctgaatagatcgcgcagctaccagagtaa

atgagcaaatgaataaatgagtagatgaattttagcggctaaaggaggcggcatggaaaatcaagaacaaccaggcacc

gacgccgtggaatgccccatgtgtggaggaacgggcggttggccaggcgtaagcggctgggttgtctgccggccctgca

atggcactggaacccccaagcccgaggaatcggcgtgacggtcgcaaaccatccggcccggtacaaatcggcgcggcgc

tgggtgatgacctggtggagaagttgaaggccgcgcaggccgcccagcggcaacgcatcgaggcagaagcacgccccgg

tgaatcgtggcaagcggccgctgatcgaatccgcaaagaatcccggcaaccgccggcagccggtgcgccgtcgattagg

aagccgcccaagggcgacgagcaaccagattttttcgttccgatgctctatgacgtgggcacccgcgatagtcgcagca

tcatggacgtggccgttttccgtctgtcgaagcgtgaccgacgagctggcgaggtgatccgctacgagcttccagacgg

gcacgtagaggtttccgcagggccggccggcatggccagtgtgtgggattacgacctggtactgatggcggtttcccat

ctaaccgaatccatgaaccgataccgggaagggaagggagacaagcccggccgcgtgttccgtccacacgttgcggacg

tactcaagttctgccggcgagccgatggcggaaagcagaaagacgacctggtagaaacctgcattcggttaaacaccac

gcacgttgccatgcagcgtacgaagaaggccaagaacggccgcctggtgacggtatccgagggtgaagccttgattagc

cgctacaagatcgtaaagagcgaaaccgggcggccggagtacatcgagatcgagctagctgattggatgtaccgcgaga

tcacagaaggcaagaacccggacgtgctgacggttcaccccgattactttttgatcgatcccggcatcggccgttttct

ctaccgcctggcacgccgcgccgcaggcaaggcagaagccagatggttgttcaagacgatctacgaacgcagtggcagc

gccggagagttcaagaagttctgtttcaccgtgcgcaagctgatcgggtcaaatgacctgccggagtacgatttgaagg

aggaggcggggcaggctggcccgatcctagtcatgcgctaccgcaacctgatcgagggcgaagcatccgccggttccta

atgtacggagcagatgctagggcaaattgccctagcaggggaaaaaggtcgaaaaggactctttcctgtggatagcacg

tacattgggaacccaaagccgtacattgggaaccggaacccgtacattgggaacccaaagccgtacattgggaaccggt

cacacatgtaagtgactgatataaaagagaaaaaaggcgatttttccgcctaaaactctttaaaacttattaaaactct

taaaacccgcctggcctgtgcataactgtctggccagcgcacagccgaagagctgcaaaaagcgcctacccttcggtcg

ctgcgctccctacgccccgccgcttcgcgtcggcctatcgcggccgctggccgctcaaaaatggctggcctacggccag

gcaatctaccagggcgcggacaagccgcgccgtcgccactcgaccgccggcgcccacatcaaggcaccctgcctcgcgc

gtttcggtgatgacggtgaaaacctctgacacatgcagctcccggtgacggtcacagcttgtctgtaagcggatgccgg

gagcagacaagcccgtcagggcgcgtcagcgggtgttggcgggtgtcggggcgcagccatgacccagtcacgtagcgat

agcggagtgtatactggcttaactatgcggcatcagagcagattgtactgagagtgcaccatatgcggtgtgaaatacc

gcacagatgcgtaaggagaaaataccgcatcaggcgctcttccgcttcctcgctcactgactcgctgcgctcggtcgtt

cggctgcggcgagcggtatcagctcactcaaaggcggtaatacggttatccacagaatcaggggataacgcaggaaaga

acatgtgagcaaaaggccagcaaaaggccaggaaccgtaaaaaggccgcgttgctggcgtttttccataggctccgccc

ccctgacgagcatcacaaaaatcgacgctcaagtcagaggtggcgaaacccgacaggactataaagataccaggcgttt

ccccctggaagctccctcgtgcgctctcctgttccgaccctgccgcttaccggatacctgtccgcctttctcccttcgg

gaagcgtggcgctttctcatagctcacgctgtaggtatctcagttcggtgtaggtcgttcgctccaagctgggctgtgt

gcacgaaccccccgttcagcccgaccgctgcgccttatccggtaactatcgtcttgagtccaacccggtaagacacgac

ttatcgccactggcagcagccactggtaacaggattagcagagcgaggtatgtaggcggtgctacagagttcttgaagt

ggtggcctaactacggctacactagaaggacagtatttggtatctgcgctctgctgaagccagttaccttcggaaaaag

agttggtagctcttgatccggcaaacaaaccaccgctggtagcggtggtttttttgtttgcaagcagcagattacgcgc

agaaaaaaaggatctcaagaagatcctttgatcttttctacggggtctgacgctcagtggaacgaaaactcacgttaag

ggattttggtcatgcattctaggtgattatttgccgactaccttggtgatctcgcctttcacgtagtggacaaattctt

ccaactgatctgcgcgcgaggccaagcgatcttcttcttgtccaagataagcctgtctagcttcaagtatgacgggctg

atactgggccggcaggcgctccattgcccagtcggcagcgacatccttcggcgcgattttgccggttactgcgctgtac

caaatgcgggacaacgtaagcactacatttcgctcatcaccagcccagtcgggcggcgagttccatagcgttaaggttt

catttagcgcctcaaatagatcctgttcaggaaccggatcaaagagttcctccgccgctggacctaccaaggcaacgct

atgttctcttgcttttgtcagcaagatagccagatcaatgtcgatcgtggctggctcgaagatacctgcaagaatgtca

ttgcgctgccattctccaaattgcagttcgcgcttagctggataacgccacggaatgatgtcgtcgtgcacaacaatgg

tgacttctacagcgcggagaatctcgctctctccaggggaagccgaagtttccaaaaggtcgttgatcaaagctcgccg

cgttgtttcatcaagccttacggtcaccgtaaccagcaaatcaatatcactgtgtggcttcaggccgccatccactgcg

gagccgtacaaatgtacggccagcaacgtcggttcgagatggcgctcgatgacgccaactacctctgatagttgagtcg

atacttcggcgatcaccgcttccctcataatgtttaactttgttttagggcgactgccctgctgcgtaacatcgttgct

gctccataacatcaaacatcgacccacggcgtaacgcgcttgctgcttggatgcccgaggcatagactgtaccccaaaa

aaacagtcataacaagccatgaaaaccgccactgcgccgttaccaccgctgcgttcggtcaaggttctggaccagttgc

gtgagcgcatacgctacttgcattacagcttacgaaccgaacaggcttatgtccactgggttcgtgccttcatccgttt

ccacggtgtgcgtcacccggcaaccttgggtagcagcgaagtcgaggcatttctgtcctggctggaacagaacttatta

tttccttcctcttttctacagtatttaaagataccccaagaagctaattataacaagacgaactccaattcactgttcc

ttgcattctaaaaccttaaataccagaaaacagctttttcaaagttgttttcaaagttggcgtataacatagtatcgac

ggagccgattttgaaaccgcggtgatcacaggcagcaacgctctgtcatcgttacaatcaacatgctaccctccgcgag

atcatccgtgtttcaaacccggcagcttagttgccgttcttccgaatagcatcggtaacatgagcaaagtctgccgcct

tacaacggctctcccgctgacgccgtcccggactgatgggctgcctgtatcgagtggtgattttgtgccgagctgccgg

tcggggagctgttggctggctggtggcaggatatattgtggtgtaaacataacggatccggtctcaggagggaggtcaa

catggtggagcacgacactctggtctactccaaaaatgtcaaagatacagtctcagaagatcaaagggctattgagact

tttcaacaaaggataatttcgggaaacctcctcggattccattgcccagctatctgtcacttcatcgaaaggacagtag

aaaaggaaggtggctcctacaaatgccatcattgcgataaaggaaaggctatcattcaagatctctctgccgacagtgg

tcccaaagatggacccccacccacgaggagcatcgtggaaaaagaagaggttccaaccacgtctacaaagcaagtggat

tgatgtgacatctccactgacgtaagggatgacgcacaatcccactatccttcgcaagacccttcctctatataaggaa

gttcatttcatttggagaggacacgctcgagtataagagctcatttttacaacaattaccaacaacaacaaacaacaaa

caacattacaattacatttacaattatcgatacaatggaacgagctatacaaggaaacgacgctagggaacaagctaac

agtgaacgttgggatggaggatcaggaggtaccacttctcccttcaaacttcctgacgaaagtccgagttggactgagt

ggcggctacataacgatgagactaattcgaatcaagataatccccttggtttcaaggaaagctggggtttcgggaaagt

tgtatttaagagatatctcagatacgacaggacggaagcttcactgcacagagtccttggatcttggacgggagattcg

gttaactatgcagcatctcgatttttcggtttcgaccagatcggatgtacctatagtattcggtttcgaggagttagta

tcaccgtttctggaggctctcgaactcttcagcatctctgtgagatggcaattcggtctaagcaagaactgctacagct

tgccccaatcgaagtggaaagtaatgtatcaagaggatgccctgaaggtactgaaaccttcgaaaaagaaagcgagtga

gcttgtcaagcagatcgttcaaacatttggcaataaagtttcttaagattgaatcctgttgccggtcttgcgatgatta

tcatataatttctgttgaattacgttaagcatgtaataattaacatgtaatgcatgacgttatttatgagatgggtttt

tatgattagagtcccgcaattatacatttaatacgcgatagaaaacaaaatatagcgcgcaaactaggataaattatcg

cgcgcggtgtcatctatgttactagatcgacgctccatggaggtgactccgaggggttgcctcaaactctatcttataa

ccggcgtggaggcatggaggcaggggtattttggtcattttaatagatagtggaaaatgacgtggaatttacttaaaga

cgaagtctttgcgacaagggggggcccacgccgaatttaatattaccggcgtggcccccccttatcgcgagtgctttag

cacgagcggtccagatttaaagtagaaaatttcccgcccactagggttaaaggtgttcacactataaaagcatatacga

tgtgatggtatttgctcgctgcttggagggaaacctcctcggattccattgcccagctatctgtcactttattgagaag

atagtggaaaaggaaggtggctcctacaaatgccatcattgcgataaaggaaaggccatcgttgaagatgcctctgccg

acagtggtcccaaagatggacccccacccacgaggagcatcgtggaaaaagaagacgttccaaccacgtcttcaaagca

agtggattgatgtgatatctccactgacgtaagggatgacgcacaatcccactatccttcgcaagacccttcctctata

taaggaagttcatttcatttggagaggtattaaaatcttaataggttttgataaaagcgaacgtggggaaacccgaacc

aaaccttcttctaaactctctctcatctctcttaaagcaaacttctctcttgtctttcttgcgtgagcgatcttcaacg

ttgtcagatcgtgcttcggcaccagtacaacgttttctttcactgaagcgaaatcaaagatctctttgtggacacgtag

tgcggcgccattaaataacgtgtacttgtcctattcttgtcggtgtggtcttgggaaaagaaagcttgctggaggctgc

tgttcagccccatacattacttgttacgattctgctgactttcggcgggtgcaatatctctacttctgcttgacgaggt

attgttgcctgtacttctttcttcttcttcttgctgattggttctataagaaatctagtattttctttgaaacagagtt

ttcccgtggttttcgaacttggagaaagattgttaagcttctgtatattctgcccaaattcgcgaccggtaatggggtc

atccaagaatgttatcaaggagttcatgaggtttaaggttcgcatggaaggaacggtcaatgggcacgagtttgaaata

gaaggcgaaggagaggggaggccatacgaaggccacaataccgtaaagcttaaggtaaccaaggggggacctttgccat

ttgcttgggatattttgtcaccacaatttcagtatggaagcaaggtatatgtcaagcaccctgccgacataccagacta

taaaaagctgtcatttcctgaaggatttaaatgggaaagggtcatgaactttgaagatggtggcgtcgttactgtaacc

caggattccagtttgcaggatggctgtttcatctacaaggtcaagttcattggcgtgaactttccttccgatggacctg

ttatgcaaaagaaaacaatgggctgggaagccagcactgagcgtttgtatcctcgtgatggcgtgttgaaaggagagat

tcataaggctctgaagctgaaagacggtggtcattacctagttgaattcaaaagtatttacatggcaaagaagcctgtg

cagctaccagggtactactatgttgactccaaactggatataacaagccacaacgaagattatacaatcgttgagcagt

atgaaagaaccgagggacgccaccatctgttcctttaagctttcccgggcatcaccatcaccatcactagctcgaggcc

tttaactctggtttcattaaattttctttagtttgaatttactgttattcggtgtgcatttctatgtttggtgagcggt

tttctgtgctcagagtgtgtttattttatgtaatttaatttctttgtgagctcctgtttagcaggtcgtcccttcagca

aggacacaaaaagattttaattttattaaaaaaaaaaaaaaaaaagaccgggaattcgatatcaagcttatcgacctgc

agatcgttcaaacatttggcaataaagtttcttaagattgaatcctgttgccggtcttgcggtgattatcatataattt

ctgttgaattacgttaagcatgtaataattaacatgtaatgcatgacgttatttatgagatgggtttttatgattagag

tcccgcaattatacatttaatacgcgatagaaaacaaaatatagcgcgcaaactaggataaattatcgcgcgcggtgtc

atctatgttactagatccgctggtaggagggaaacctcctcggattccattgcccagctatctgtcactttattgagaa

gatagtggaaaaggaaggtggctcctacaaatgccatcattgcgataaaggaaaggccatcgttgaagatgcctctgcc

gacagtggtcccaaagatggacccccacccacgaggagcatcgtggaaaaagaagacgttccaaccacgtcttcaaagc

aagtggattgatgtgatatctccactgacgtaagggatgacgcacaatcccactatccttcgcaagacccttcctctat

ataaggaagttcatttcatttggagaggtattaaaatcttaataggttttgataaaagcgaacgtggggaaacccgaac

caaaccttcttctaaactctctctcatctctcttaaagcaaacttctctcttgtctttcttgcgtgagcgatcttcaac

gttgtcagatcgtgcttcggcaccagtacaacgttttctttcactgaagcgaaatcaaagatctctttgtggacacgta

gtgcggcgccattaaataacgtgtacttgtcctattcttgtcggtgtggtcttgggaaaagaaagcttgctggaggctg

ctgttcagccccatacattacttgttacgattctgctgactttcggcgggtgcaatatctctacttctgcttgacgagg

tattgttgcctgtacttctttcttcttcttcttgctgattggttctataagaaatctagtattttctttgaaacagagt

tttcccgtggttttcgaacttggagaaagattgttaagcttctgtatattctgcccaaattcgcgaccggtaatggtga

gcaagggcgaggagctgttcaccggggtggtgcccatcctggtcgagctggacggcgacgtaaacggccacaagttcag

cgtgtccggcgagggcgagggcgatgccacctacggcaagctgaccctgaagttcatctgcaccaccggcaagctgccc

gtgccctggcccaccctcgtgaccaccttcagctacggcgtgcagtgcttcagccgctaccccgaccacatgaagcagc

acgacttcttcaagtccgccatgcccgaaggctacgtccaggagcgcaccatcttcttcaaggacgacggcaactacaa

gacccgcgccgaggtgaagttcgagggcgacaccctggtgaaccgcatcgagctgaagggcatcgacttcaaggaggac

ggcaacatcctggggcacaagctggagtacaactacaacagccacaacgtctatatcatggccgacaagcagaagaacg

gcatcaaggtgaacttcaagatccgccacaacatcgaggacggcagcgtgcagctcgccgaccactaccagcagaacac

ccccatcggcgacggccccgtgctgctgcccgacaaccactacctgagcacccagtccgccctgagcaaagaccccaac

gagaagcgcgatcacatggtcctgctggagttcgtgaccgccgccgggatcactcacggcatggacgagctgtacaagt

aagctttcccgggcatcaccatcaccatcactagctcgaggcctttaactctggtttcattaaattttctttagtttga

atttactgttattcggtgtgcatttctatgtttggtgagcggttttctgtgctcagagtgtgtttattttatgtaattt

aatttctttgtgagctcctgtttagcaggtcgtcccttcagcaaggacacaaaaagattttaattttattaaaaaaaaa

aaaaaaaaagaccgggaattcgatatcaagcttatcgacctgcagatcgttcaaacatttggcaataaagtttcttaag

attgaatcctgttgccggtcttgcggtgattatcatataatttctgttgaattacgttaagcatgtaataattaacatg

taatgcatgacgttatttatgagatgggtttttatgattagagtcccgcaattatacatttaatacgcgatagaaaaca

aaatatagcgcgcaaactaggataaattatcgcgcgcggtgtcatctatgttactagatccgctgtcaagcgaatgatt

attttatgaatatatttcattgtgcaagtagatagaaattacatatgttacataacacacgaaataaacaaaaaaagac

aatccaaaaacaaacaccccaaaaaaaataatcactttagataaactcgtatgaggagaggcacgttaagctcagtgac

tcgacgattcccgagcaaaaaaagtctccccgtcacacatatagtgggtgacgcaattatctttaaagtaatccttctg

ttgacttgtcattgataacatccagtcttcgtcaggattgcaaagaattatagaagggatcccaccttttattttcttc

ttttttccatatttagggttgacagtgaaatcagactggcaacctattaattgcttccacaatgggacgaacttgaagg

ggatgtcgtcgatgatattataggtggcgtgttcatcgtagttggtgaaatcgatggtaccgttccaatagttgtgtcg

tccgagacttctagcccaggtggtctttccggtacgagttggtccgcagatgtagaggctggggtgtcggattccattc

cttccattgtcctggttaaatcggccatccattcaaggtcagattgagcttgttggtatgagacaggatgtatgtaagt

ataagcgtctatgcttacatggtatagatgggtttccctccaggagtgtagatcttcgtggcagcgaagatctgattct

gtgaagggcgacacatacggttcaggttgtggagggaataatttgttggctgaatattccagccattgaagttttgttg

cccattcatgagggaattcttccttgatcatgtcaagatattcctccttagacgttgcagtctggataatagttctcca

tcgtgcgtcagatttgcgaggagataccttatgatctcggaaatctcctctggttttaatatctccgtcctttgatatg

taatcaaggacttgtttagagtttctagctggctggatattagggtgatttccttcaaaatcgaaaaaagaaggatccc

taatacaaggttttttatcaagctggagaagagcatgatagtgggtagtgccatcttgatgaagctcagaagcaacacc

aaggaagaaaataagaaaaggtgtgagtttctcccagagaaactggaataaatcatctctttgagatgagcacttggga

taggtaaggaaaacatatttagattggagtctgaagttcttactagcagaaggcattttgttgtgactccgaggggttg

cctcaaactctatcttataaccggcgtggaggcatggaggcaggggtattttggtcattttaatagatagtggaaaatg

acgtggaatttacttaaagacgaagtctttgcgacaagggggggcccacgccgaatttaatattaccggcgtggccccc

ccttatcgcgagtgctttagcacgagcggtccagatttaaagtagaaaatttcccgcccactagggttaaaggtgttca

cactatacaagcatatacgatgtgatggtattgactagagtttctccgct
